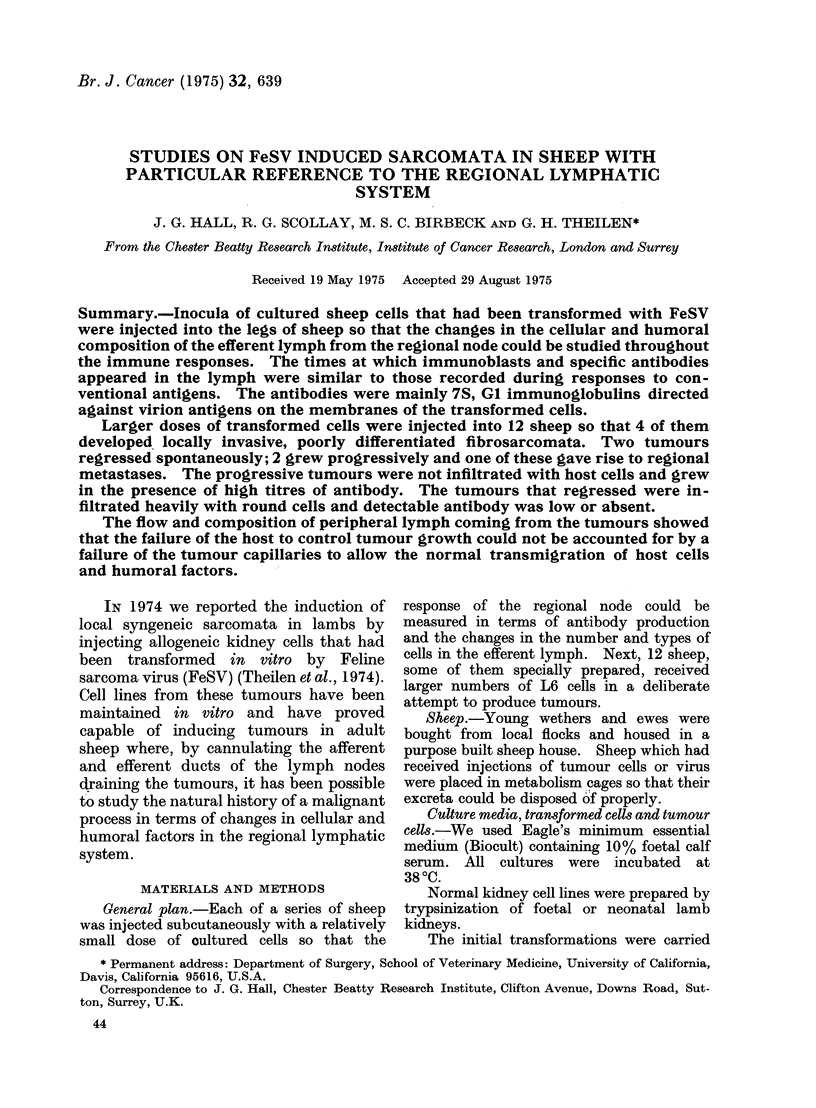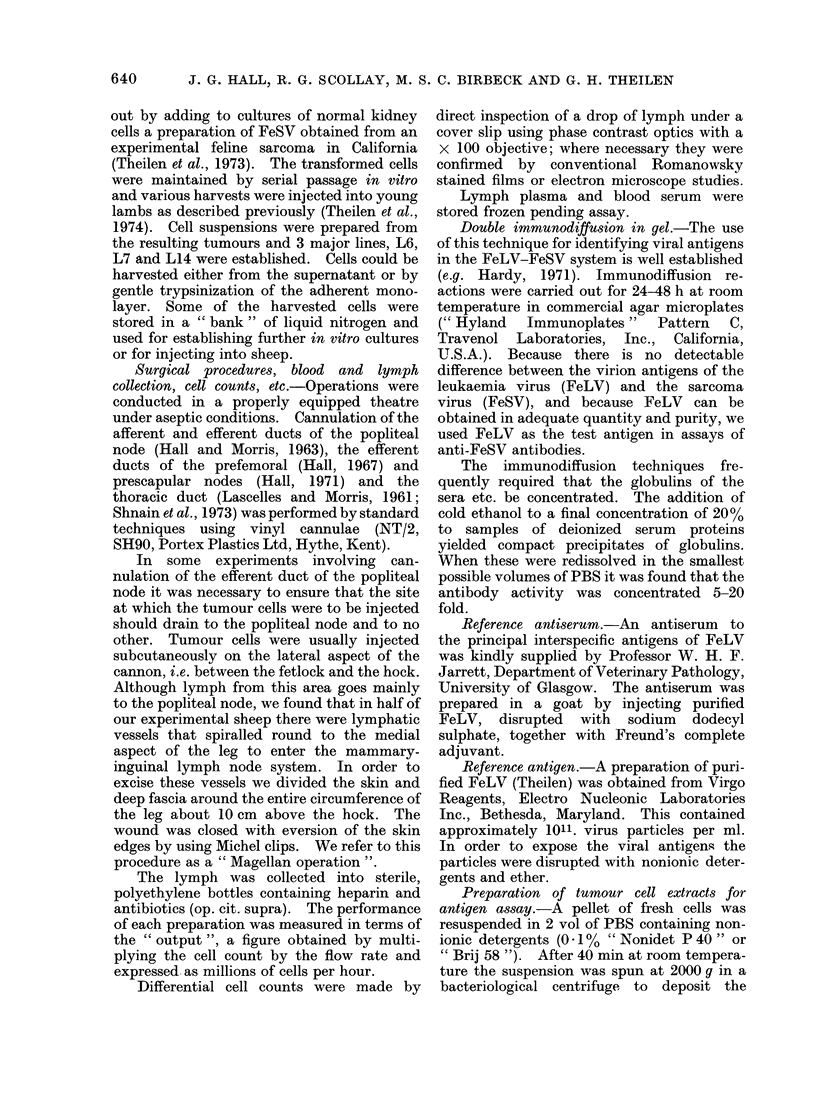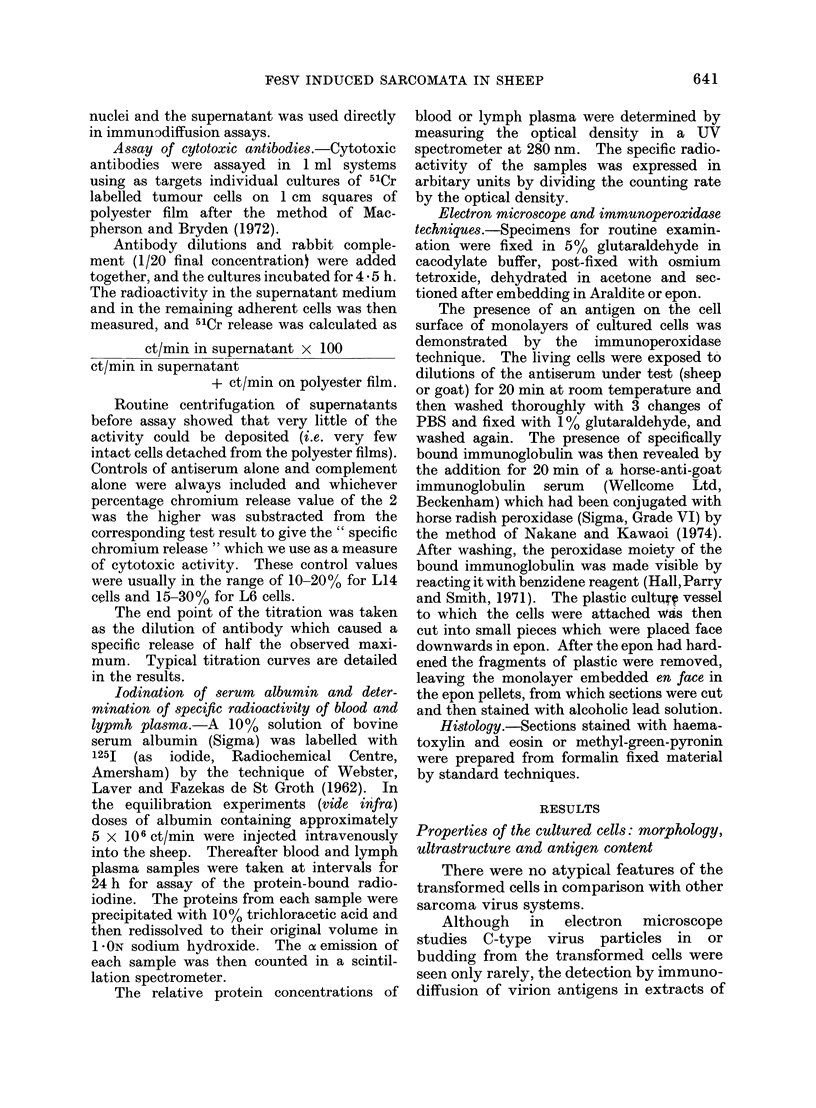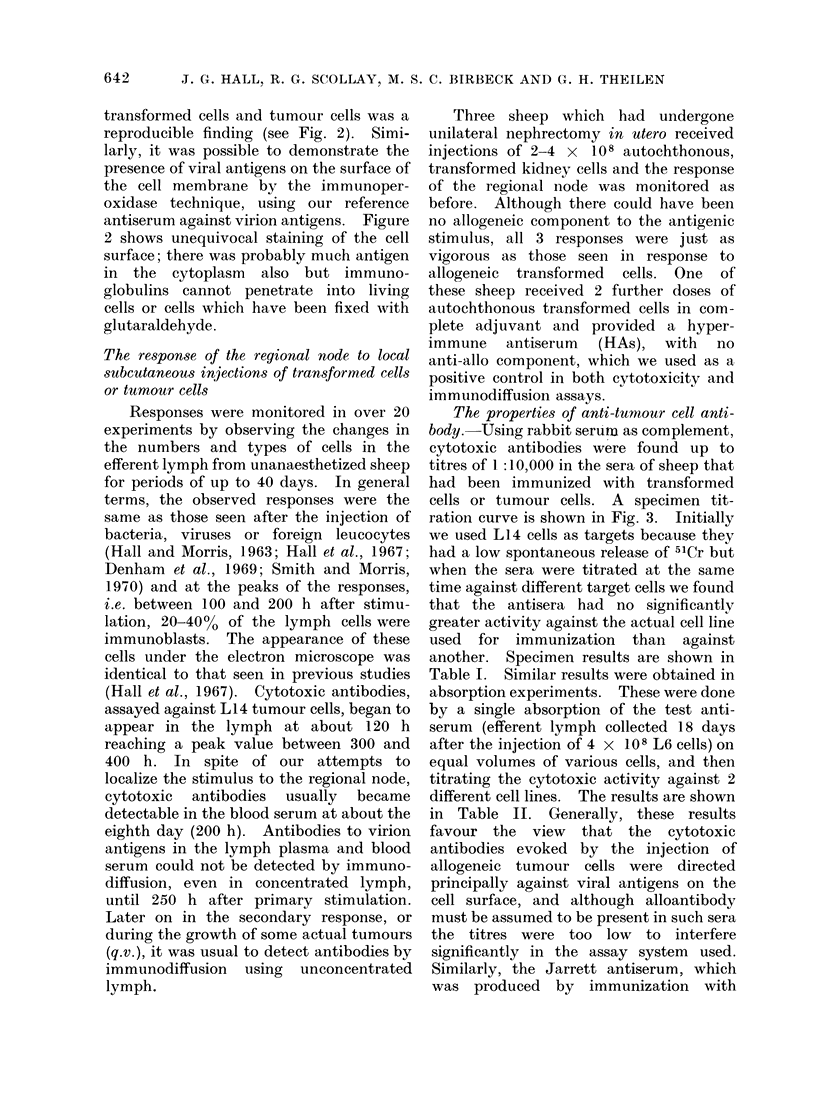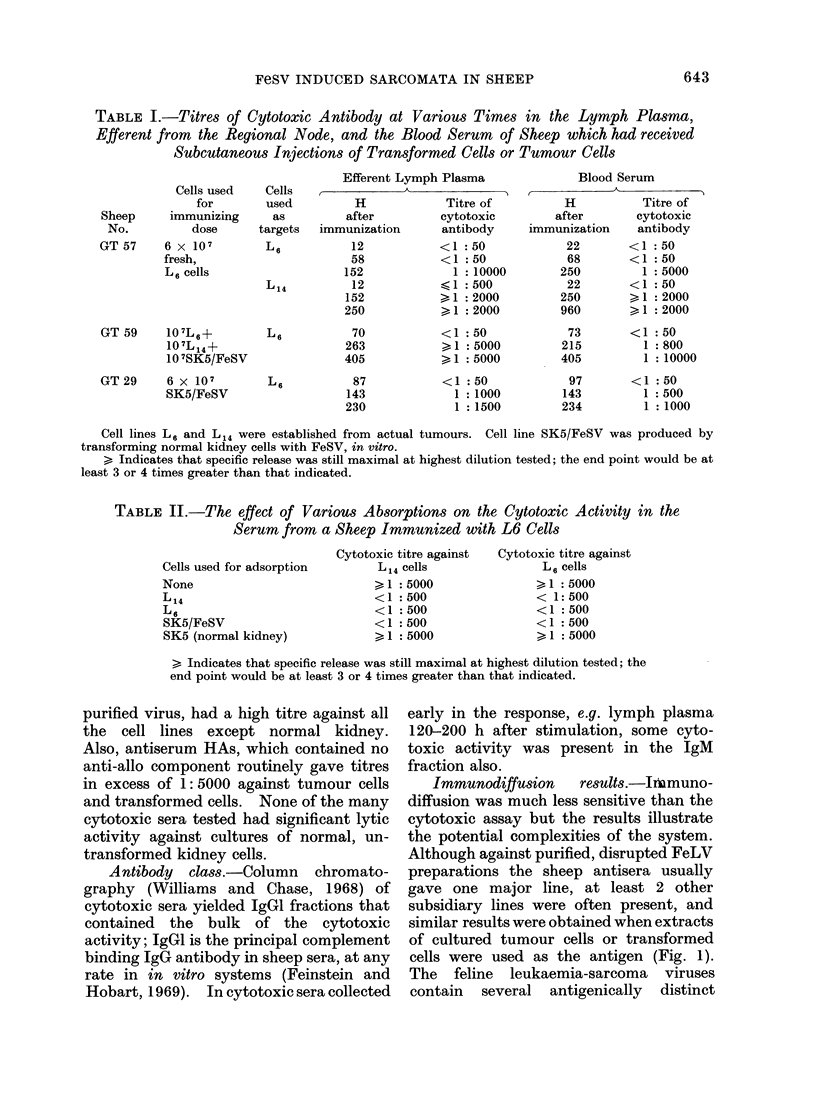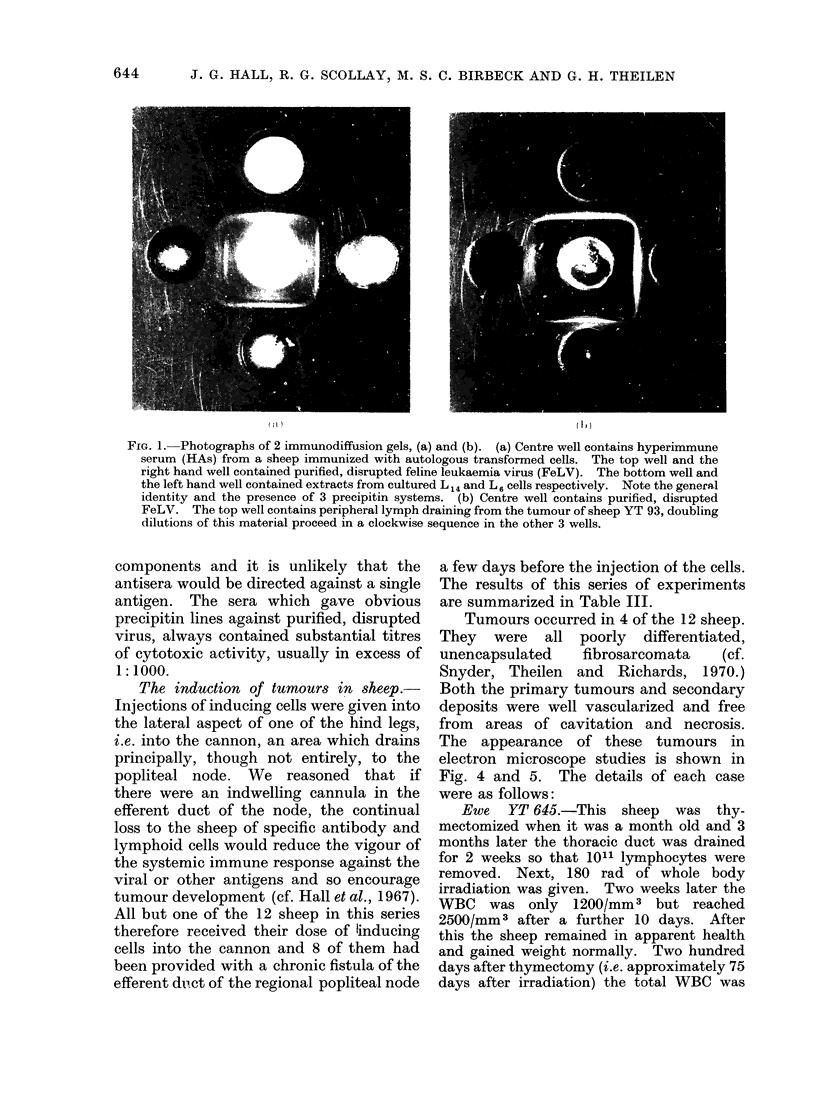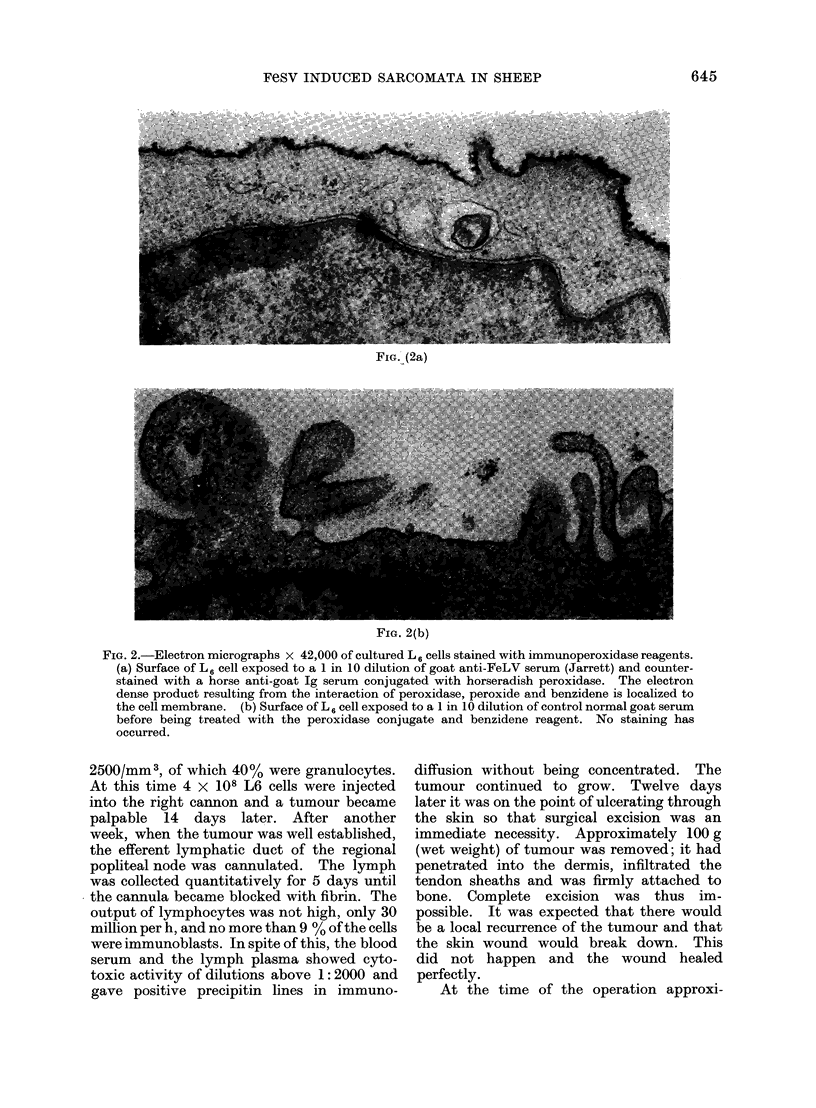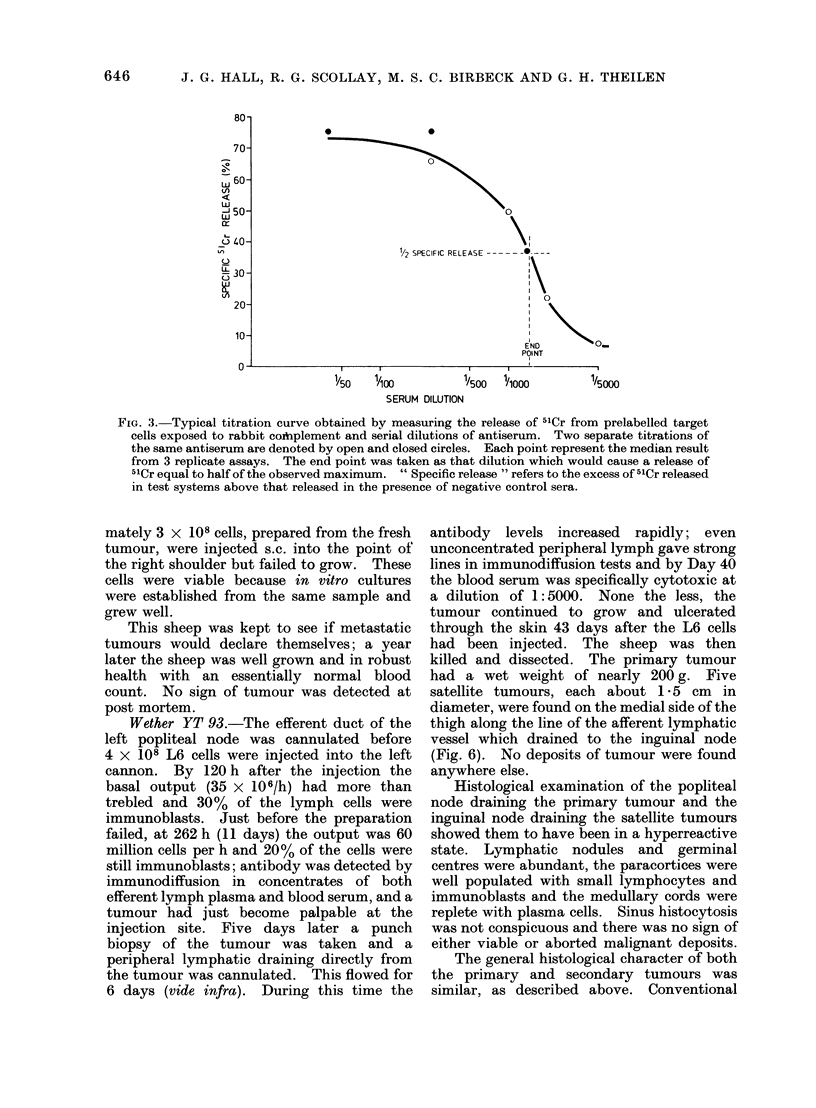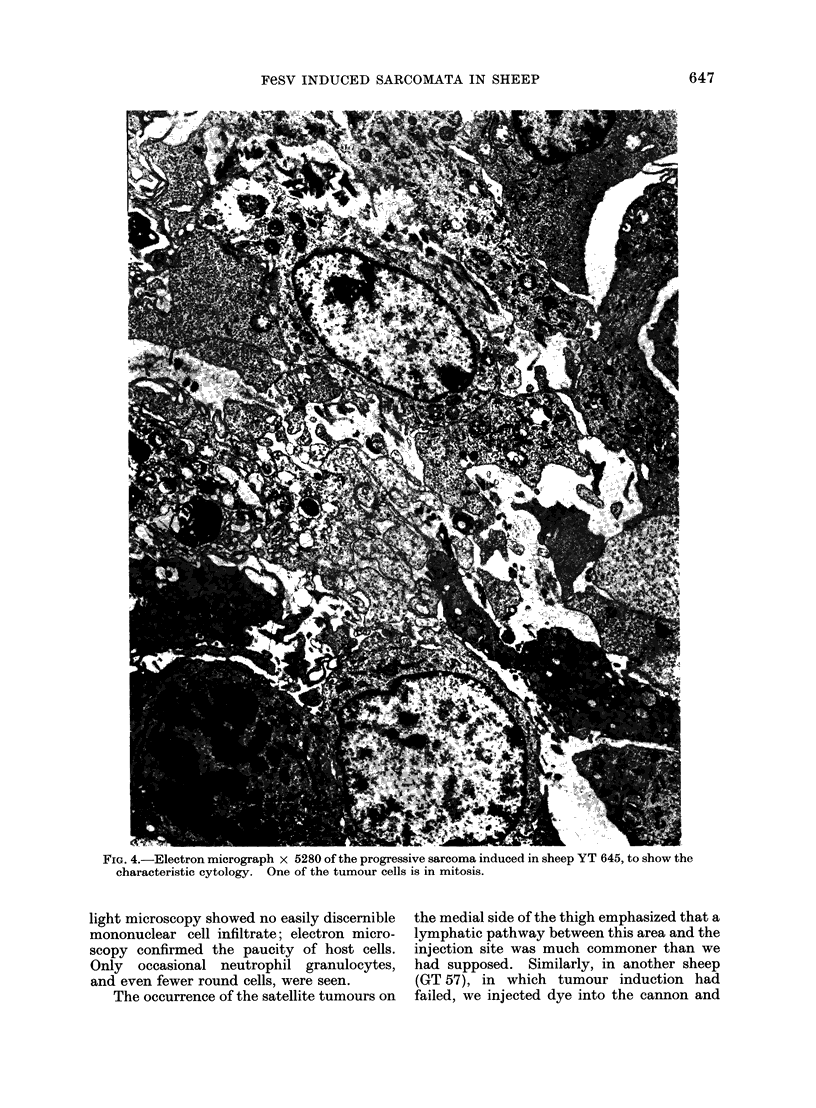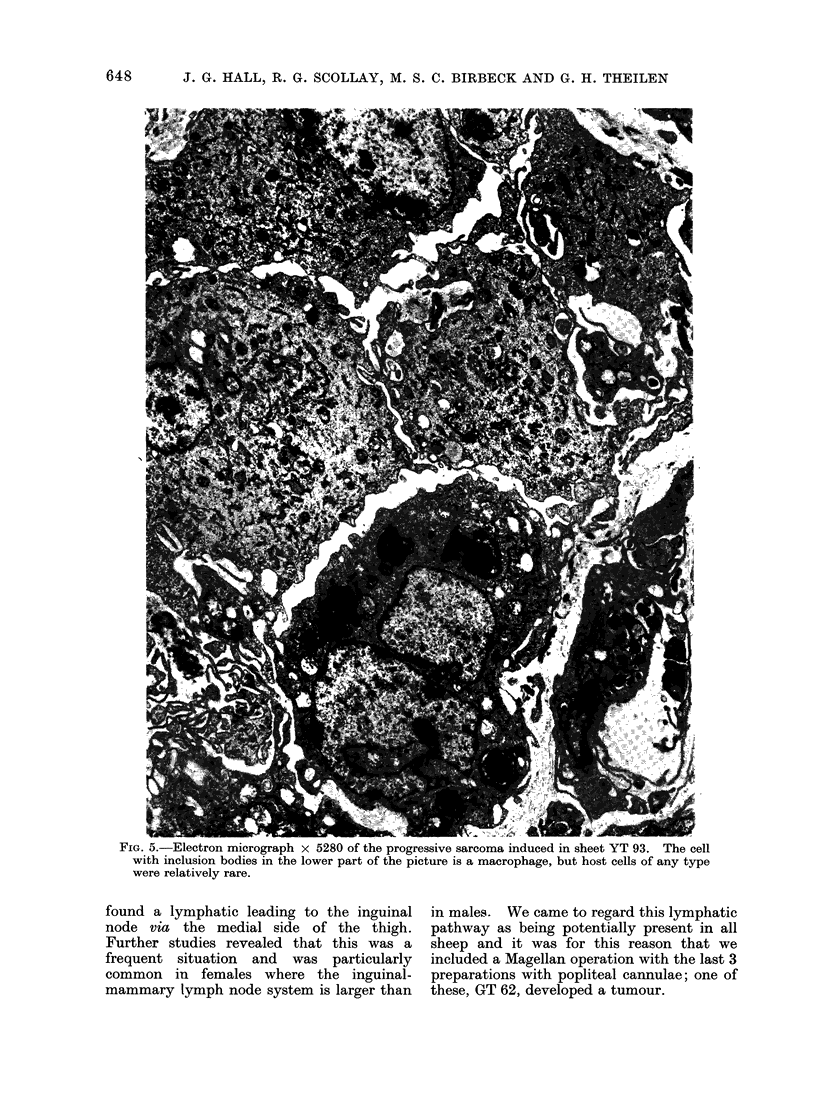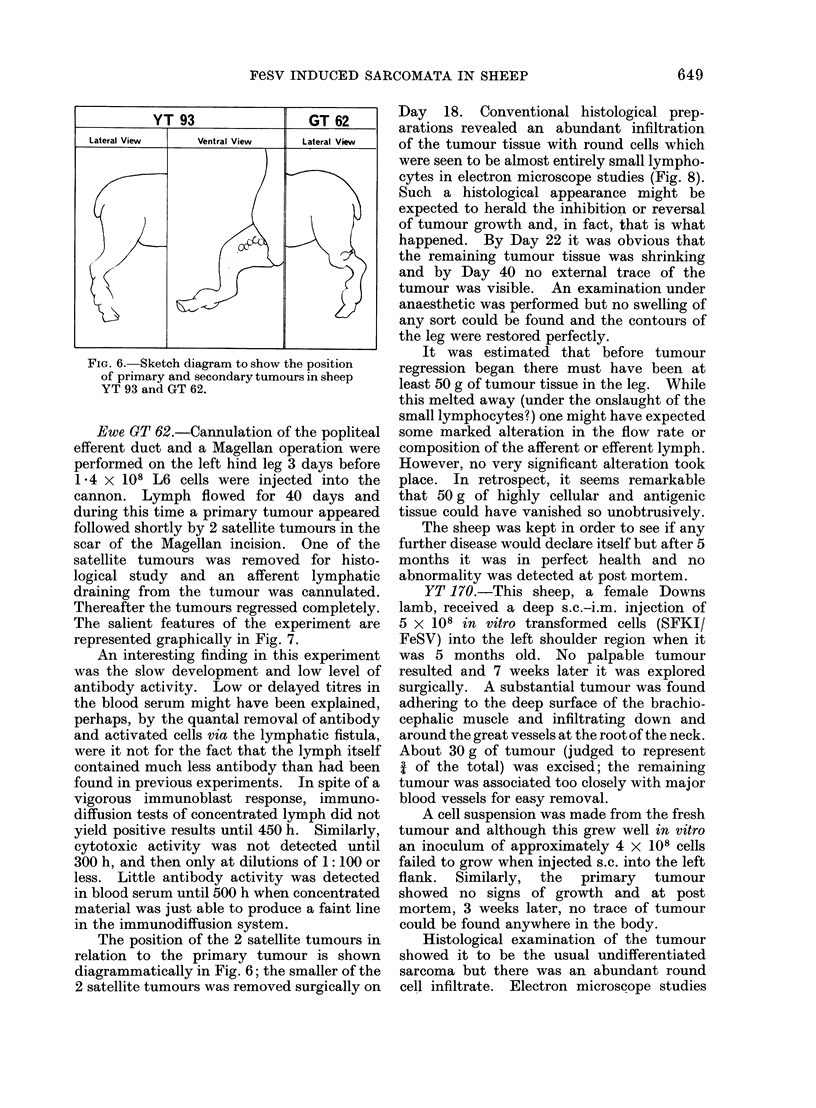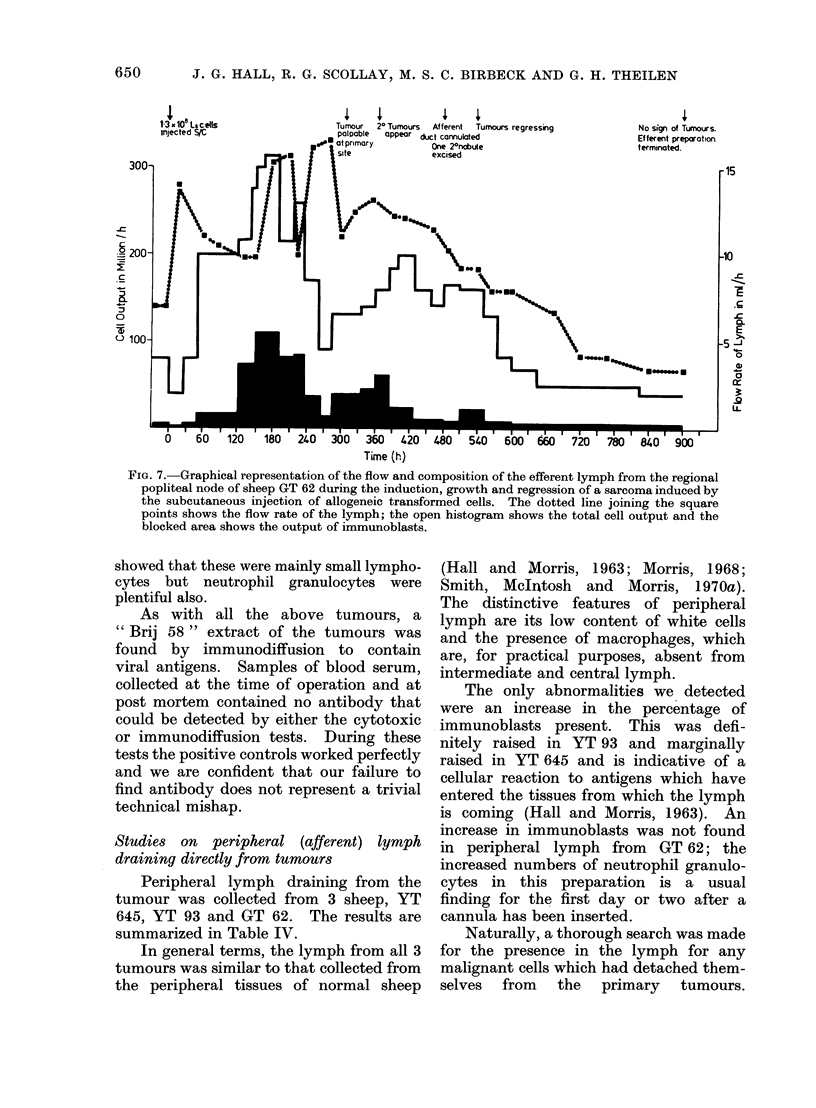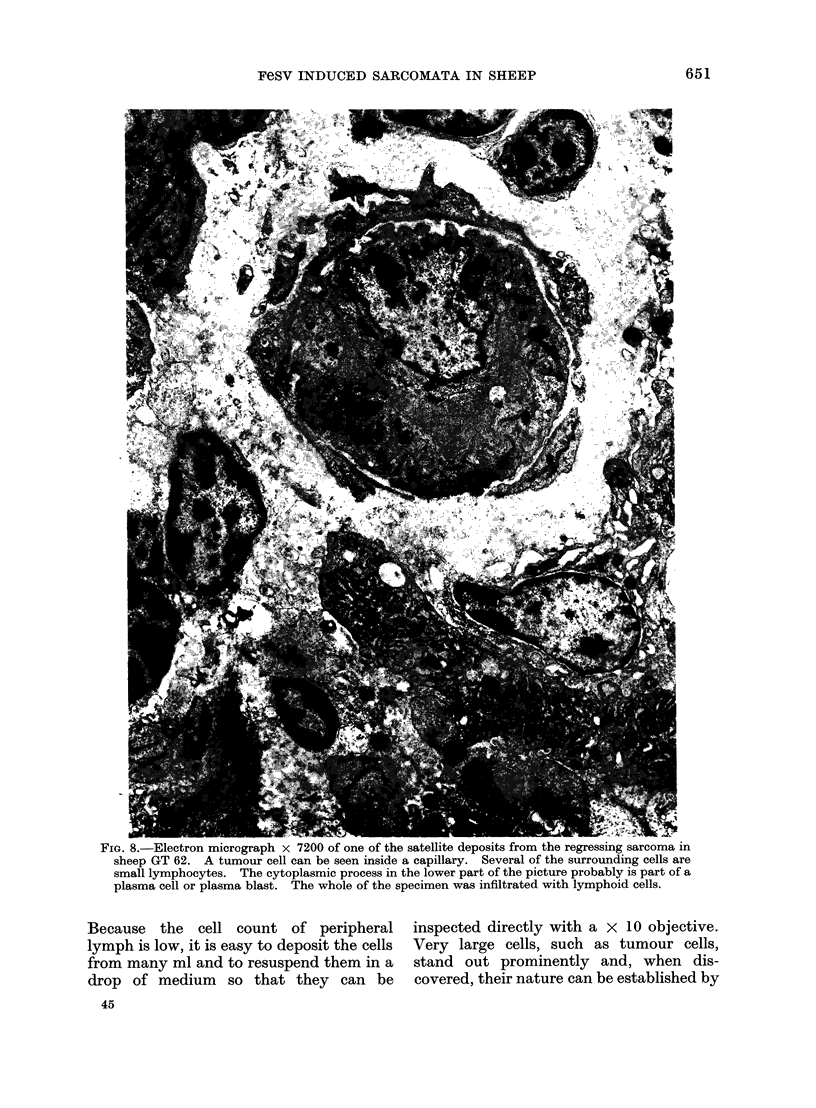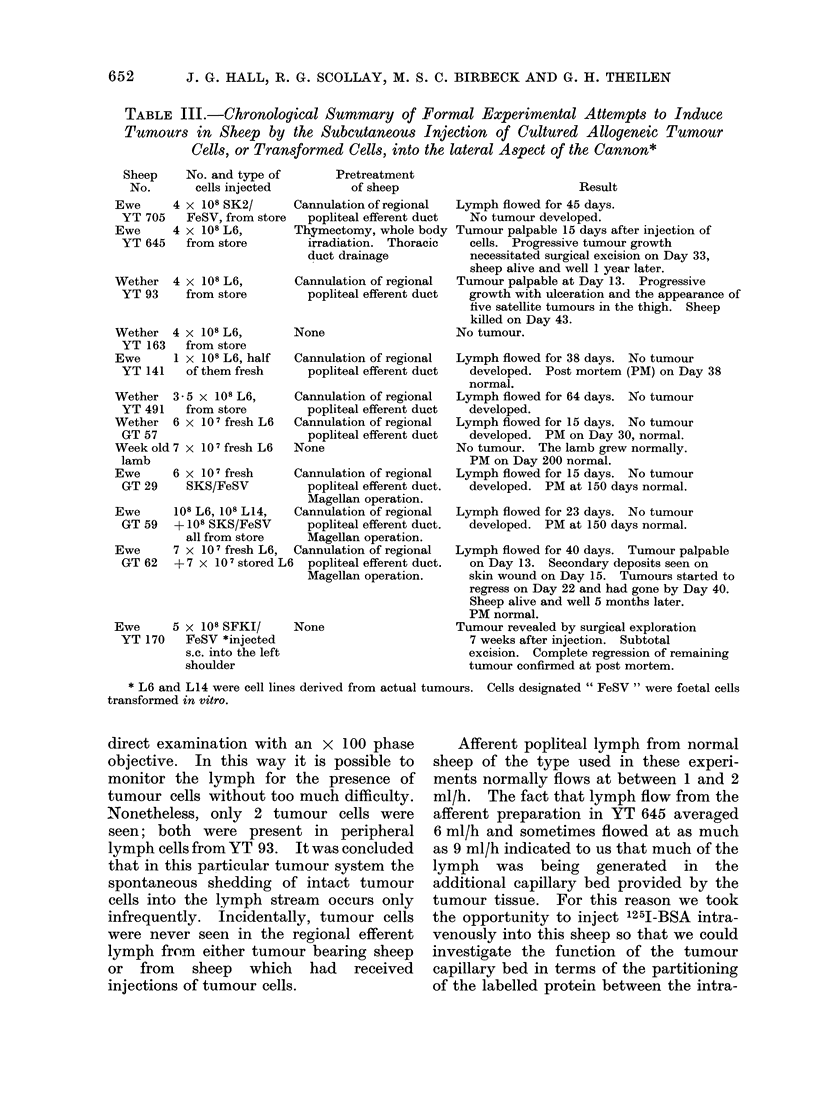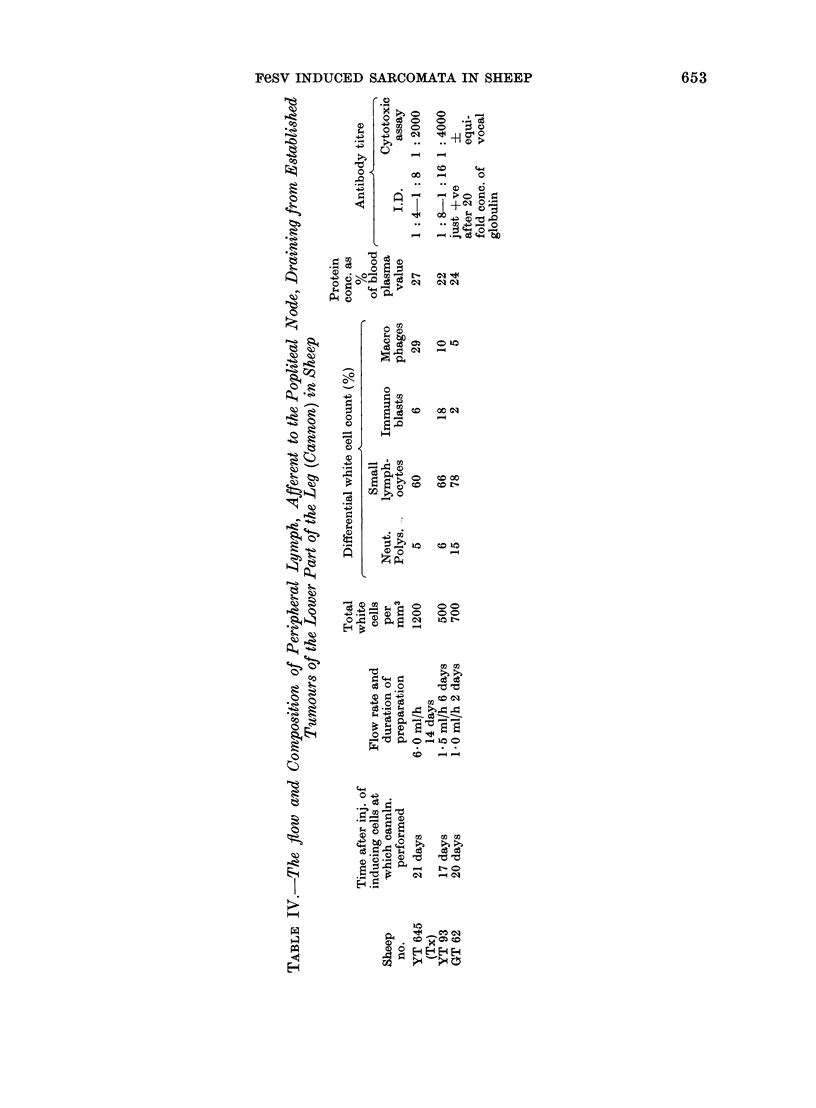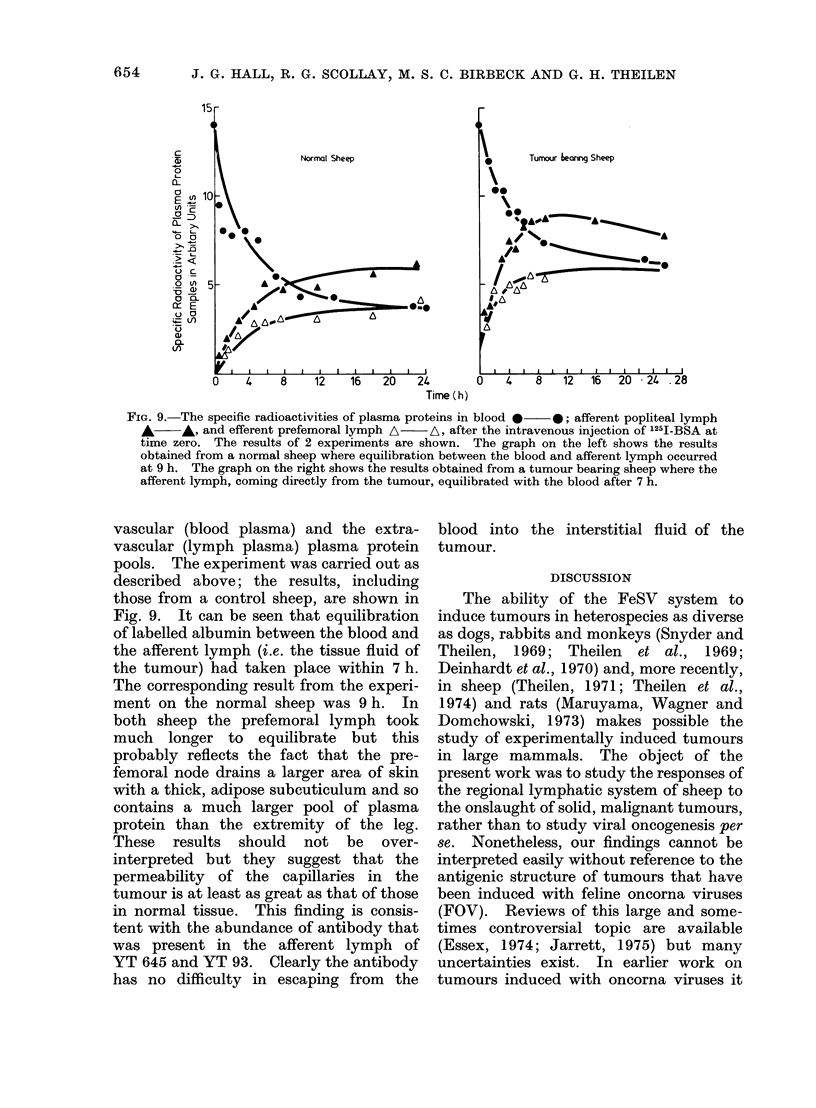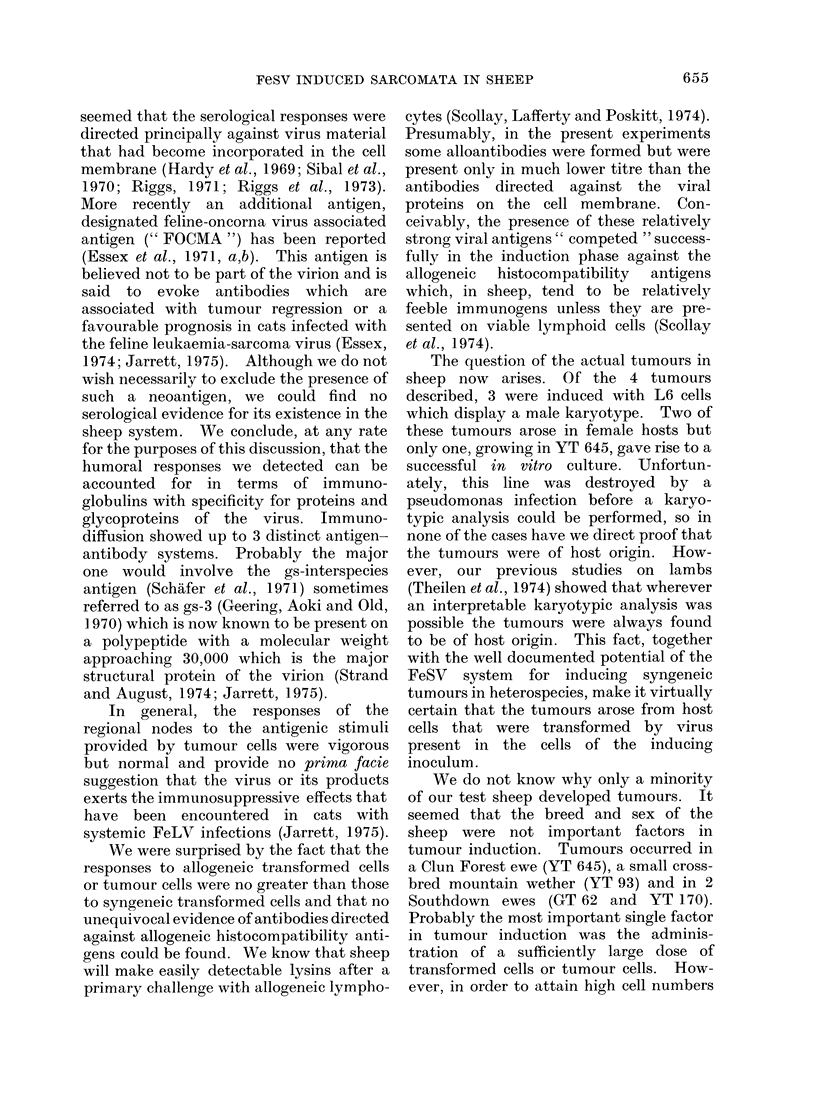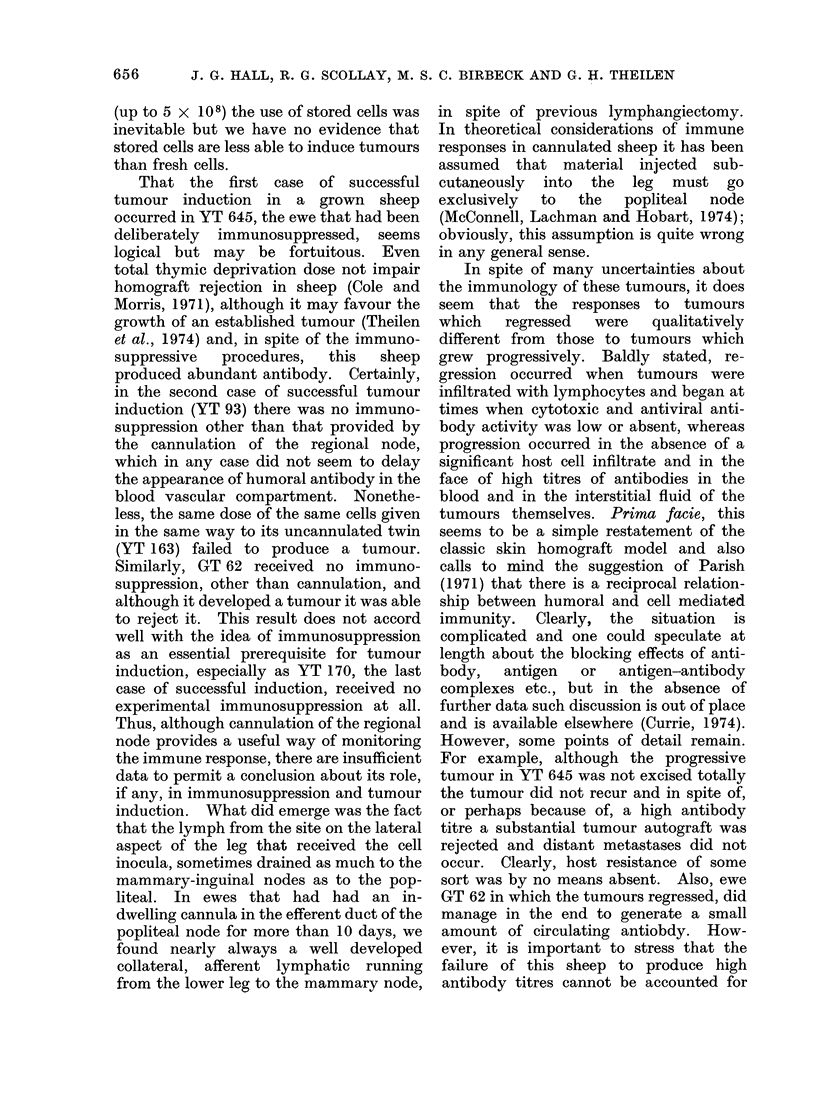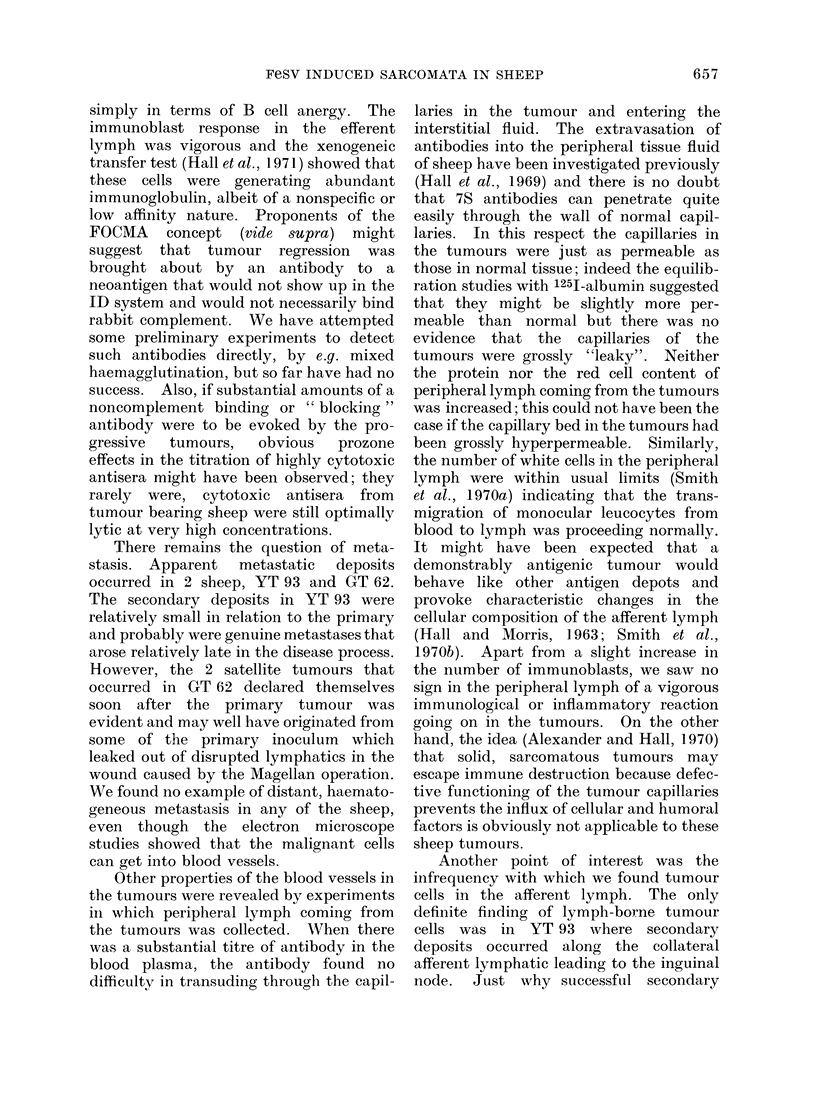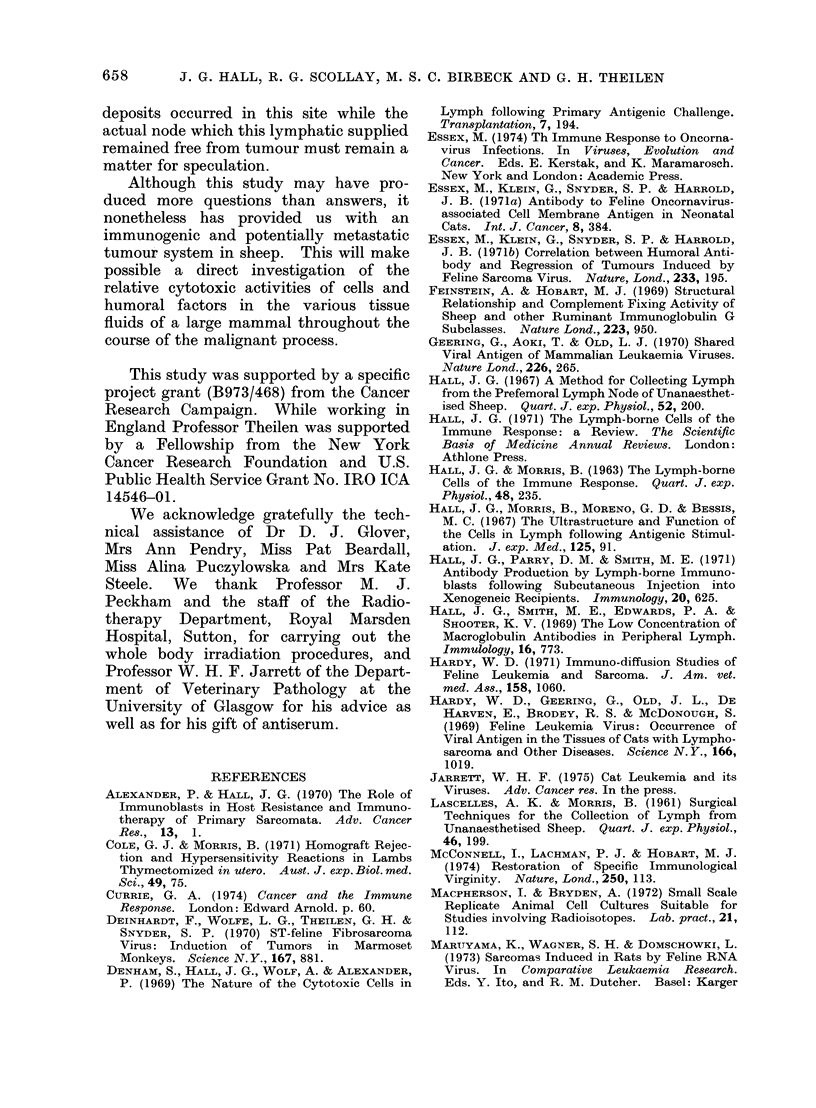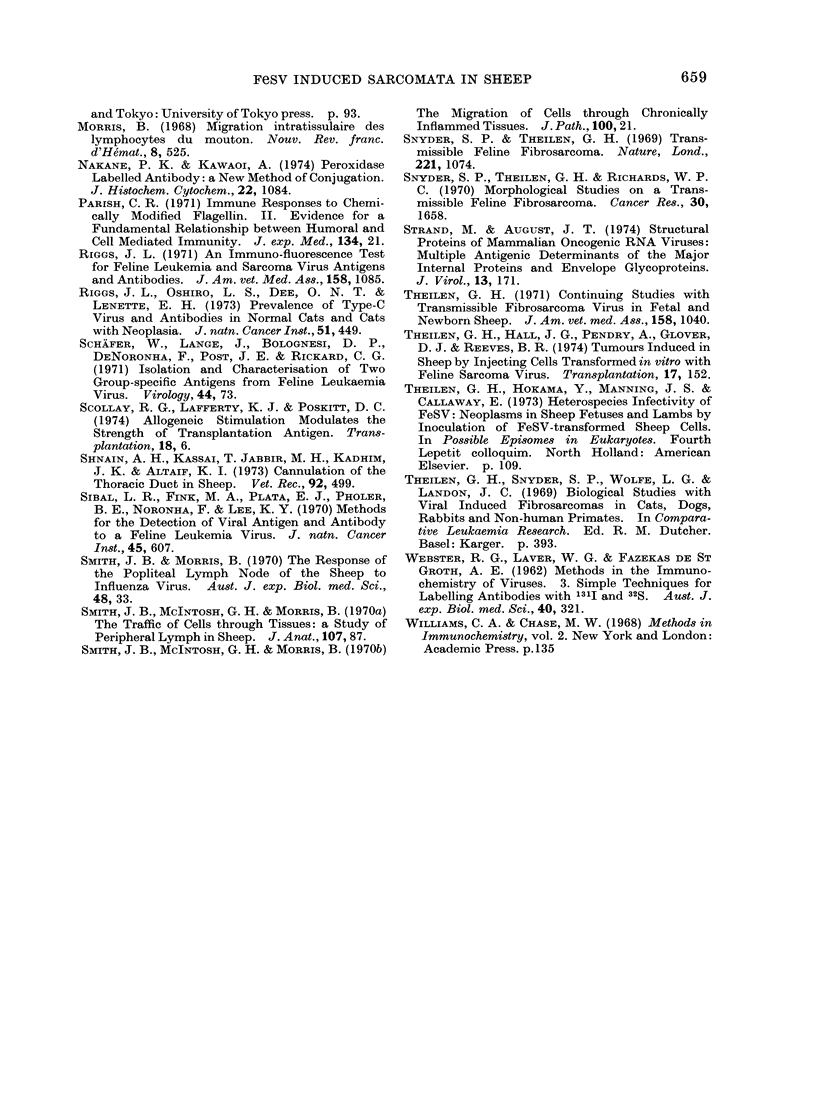# Studies on FeSV induced sarcomata in sheep with particular reference to the regional lymphatic system.

**DOI:** 10.1038/bjc.1975.275

**Published:** 1975-12

**Authors:** J. G. Hall, R. G. Scollay, M. S. Birbeck, G. H. Theilen

## Abstract

**Images:**


*Br. J. Cancer* (1975) **32**, 639

### STUDIES ON FeSV INDUCED SARCOMATA IN SHEEP WITH PARTICULAR REFERENCE TO THE REGIONAL LYMPHATIC SYSTEM

J. G. HALL, R. G. SCOLLAY, M. S. C. BIRBECK AND G. H. THEILEN*

*From the Chester Beatty Research Institute, Institute of Cancer Research, London and Surrey*

Received 19 May 1975 Accepted 29 August 1975

**Summary.—Inocula of cultured sheep cells that had been transformed with FeSV were injected into the legs of sheep so that the changes in the cellular and humoral composition of the efferent lymph from the regional node could be studied throughout the immune responses. The times at which immunoblasts and specific antibodies appeared in the lymph were similar to those recorded during responses to conventional antigens. The antibodies were mainly 7S, G1 immunoglobulins directed against virion antigens on the membranes of the transformed cells.**

**Larger doses of transformed cells were injected into 12 sheep so that 4 of them developed locally invasive, poorly differentiated fibrosarcomata. Two tumours regressed spontaneously; 2 grew progressively and one of these gave rise to regional metastases. The progressive tumours were not infiltrated with host cells and grew in the presence of high titres of antibody. The tumours that regressed were infiltrated heavily with round cells and detectable antibody was low or absent.**

**The flow and composition of peripheral lymph coming from the tumours showed that the failure of the host to control tumour growth could not be accounted for by a failure of the tumour capillaries to allow the normal transmigration of host cells and humoral factors.**

IN 1974 we reported the induction of local syngeneic sarcomata in lambs by injecting allogeneic kidney cells that had been transformed *in vitro* by Feline sarcoma virus (FeSV) (Theilen *et al.*, 1974). Cell lines from these tumours have been maintained *in vitro* and have proved capable of inducing tumours in adult sheep where, by cannulating the afferent and efferent ducts of the lymph nodes draining the tumours, it has been possible to study the natural history of a malignant process in terms of changes in cellular and humoral factors in the regional lymphatic system.

### MATERIALS AND METHODS

*General plan.*—Each of a series of sheep was injected subcutaneously with a relatively small dose of cultured cells so that the response of the regional node could be measured in terms of antibody production and the changes in the number and types of cells in the efferent lymph. Next, 12 sheep, some of them specially prepared, received larger numbers of L6 cells in a deliberate attempt to produce tumours.

*Sheep.*—Young wethers and ewes were bought from local flocks and housed in a purpose built sheep house. Sheep which had received injections of tumour cells or virus were placed in metabolism cages so that their excreta could be disposed of properly.

*Culture media, transformed cells and tumour cells.*—We used Eagle's minimum essential medium (Biocult) containing 10% foetal calf serum. All cultures were incubated at 38 °C.

Normal kidney cell lines were prepared by trypsinization of foetal or neonatal lamb kidneys.

The initial transformations were carried

* Permanent address: Department of Surgery, School of Veterinary Medicine, University of California, Davis, California 95616, U.S.A.

Correspondence to J. G. Hall, Chester Beatty Research Institute, Clifton Avenue, Downs Road, Sutton, Surrey, U.K.

out by adding to cultures of normal kidney cells a preparation of FeSV obtained from an experimental feline sarcoma in California (Theilen *et al.*, 1973). The transformed cells were maintained by serial passage *in vitro* and various harvests were injected into young lambs as described previously (Theilen *et al.*, 1974). Cell suspensions were prepared from the resulting tumours and 3 major lines, L6, L7 and L14 were established. Cells could be harvested either from the supernatant or by gentle trypsinization of the adherent monolayer. Some of the harvested cells were stored in a " bank " of liquid nitrogen and used for establishing further *in vitro* cultures or for injecting into sheep.

*Surgical procedures, blood and lymph collection, cell counts, etc.*—Operations were conducted in a properly equipped theatre under aseptic conditions. Cannulation of the afferent and efferent ducts of the popliteal node (Hall and Morris, 1963), the efferent ducts of the prefemoral (Hall, 1967) and prescapular nodes (Hall, 1971) and the thoracic duct (Lascelles and Morris, 1961; Shnain *et al.*, 1973) was performed by standard techniques using vinyl cannulae (NT/2, SH90, Portex Plastics Ltd, Hythe, Kent).

In some experiments involving cannulation of the efferent duct of the popliteal node it was necessary to ensure that the site at which the tumour cells were to be injected should drain to the popliteal node and to no other. Tumour cells were usually injected subcutaneously on the lateral aspect of the cannon, *i.e.* between the fetlock and the hock. Although lymph from this area goes mainly to the popliteal node, we found that in half of our experimental sheep there were lymphatic vessels that spiralled round to the medial aspect of the leg to enter the mammary-inguinal lymph node system. In order to excise these vessels we divided the skin and deep fascia around the entire circumference of the leg about 10 cm above the hock. The wound was closed with eversion of the skin edges by using Michel clips. We refer to this procedure as a " Magellan operation ".

The lymph was collected into sterile, polyethylene bottles containing heparin and antibiotics (op. cit. supra). The performance of each preparation was measured in terms of the " output ", a figure obtained by multiplying the cell count by the flow rate and expressed as millions of cells per hour.

Differential cell counts were made by direct inspection of a drop of lymph under a cover slip using phase contrast optics with a $\times$ 100 objective; where necessary they were confirmed by conventional Romanowsky stained films or electron microscope studies.

Lymph plasma and blood serum were stored frozen pending assay.

*Double immunodiffusion in gel.*—The use of this technique for identifying viral antigens in the FeLV–FeSV system is well established (*e.g.* Hardy, 1971). Immunodiffusion reactions were carried out for 24–48 h at room temperature in commercial agar microplates (" Hyland Immunoplates " Pattern C, Travenol Laboratories, Inc., California, U.S.A.). Because there is no detectable difference between the virion antigens of the leukaemia virus (FeLV) and the sarcoma virus (FeSV), and because FeLV can be obtained in adequate quantity and purity, we used FeLV as the test antigen in assays of anti-FeSV antibodies.

The immunodiffusion techniques frequently required that the globulins of the sera etc. be concentrated. The addition of cold ethanol to a final concentration of 20% to samples of deionized serum proteins yielded compact precipitates of globulins. When these were redissolved in the smallest possible volumes of PBS it was found that the antibody activity was concentrated 5–20 fold.

*Reference antiserum.*—An antiserum to the principal interspecific antigens of FeLV was kindly supplied by Professor W. H. F. Jarrett, Department of Veterinary Pathology, University of Glasgow. The antiserum was prepared in a goat by injecting purified FeLV, disrupted with sodium dodecyl sulphate, together with Freund's complete adjuvant.

*Reference antigen.*—A preparation of purified FeLV (Theilen) was obtained from Virgo Reagents, Electro Nucleonic Laboratories Inc., Bethesda, Maryland. This contained approximately $10^{11}$. virus particles per ml. In order to expose the viral antigens the particles were disrupted with nonionic detergents and ether.

*Preparation of tumour cell extracts for antigen assay.*—A pellet of fresh cells was resuspended in 2 vol of PBS containing nonionic detergents (0·1% " Nonidet P 40 " or " Brij 58 "). After 40 min at room temperature the suspension was spun at 2000 *g* in a bacteriological centrifuge to deposit the nuclei and the supernatant was used directly in immunodiffusion assays.

*Assay of cytotoxic antibodies.*—Cytotoxic antibodies were assayed in 1 ml systems using as targets individual cultures of $^{51}Cr$ labelled tumour cells on 1 cm squares of polyester film after the method of Macpherson and Bryden (1972).

Antibody dilutions and rabbit complement (1/20 final concentration) were added together, and the cultures incubated for 4·5 h. The radioactivity in the supernatant medium and in the remaining adherent cells was then measured, and $^{51}Cr$ release was calculated as

$$\frac{\text{ct/min in supernatant} \times 100}{\text{ct/min in supernatant} + \text{ct/min on polyester film.}}$$

Routine centrifugation of supernatants before assay showed that very little of the activity could be deposited (*i.e.* very few intact cells detached from the polyester films). Controls of antiserum alone and complement alone were always included and whichever percentage chromium release value of the 2 was the higher was substracted from the corresponding test result to give the " specific chromium release " which we use as a measure of cytotoxic activity. These control values were usually in the range of 10–20% for L14 cells and 15–30% for L6 cells.

The end point of the titration was taken as the dilution of antibody which caused a specific release of half the observed maximum. Typical titration curves are detailed in the results.

*Iodination of serum albumin and determination of specific radioactivity of blood and lypmh plasma.*—A 10% solution of bovine serum albumin (Sigma) was labelled with $^{125}I$ (as iodide, Radiochemical Centre, Amersham) by the technique of Webster, Laver and Fazekas de St Groth (1962). In the equilibration experiments (*vide infra*) doses of albumin containing approximately $5 \times 10^6$ ct/min were injected intravenously into the sheep. Thereafter blood and lymph plasma samples were taken at intervals for 24 h for assay of the protein-bound radioiodine. The proteins from each sample were precipitated with 10% trichloracetic acid and then redissolved to their original volume in 1·0N sodium hydroxide. The α emission of each sample was then counted in a scintillation spectrometer.

The relative protein concentrations of blood or lymph plasma were determined by measuring the optical density in a UV spectrometer at 280 nm. The specific radioactivity of the samples was expressed in arbitary units by dividing the counting rate by the optical density.

*Electron microscope and immunoperoxidase techniques.*—Specimens for routine examination were fixed in 5% glutaraldehyde in cacodylate buffer, post-fixed with osmium tetroxide, dehydrated in acetone and sectioned after embedding in Araldite or epon.

The presence of an antigen on the cell surface of monolayers of cultured cells was demonstrated by the immunoperoxidase technique. The living cells were exposed to dilutions of the antiserum under test (sheep or goat) for 20 min at room temperature and then washed thoroughly with 3 changes of PBS and fixed with 1% glutaraldehyde, and washed again. The presence of specifically bound immunoglobulin was then revealed by the addition for 20 min of a horse-anti-goat immunoglobulin serum (Wellcome Ltd, Beckenham) which had been conjugated with horse radish peroxidase (Sigma, Grade VI) by the method of Nakane and Kawaoi (1974). After washing, the peroxidase moiety of the bound immunoglobulin was made visible by reacting it with benzidene reagent (Hall, Parry and Smith, 1971). The plastic culture vessel to which the cells were attached was then cut into small pieces which were placed face downwards in epon. After the epon had hardened the fragments of plastic were removed, leaving the monolayer embedded *en face* in the epon pellets, from which sections were cut and then stained with alcoholic lead solution.

*Histology.*—Sections stained with haematoxylin and eosin or methyl-green-pyronin were prepared from formalin fixed material by standard techniques.

### RESULTS

### *Properties of the cultured cells: morphology, ultrastructure and antigen content*

There were no atypical features of the transformed cells in comparison with other sarcoma virus systems.

Although in electron microscope studies C-type virus particles in or budding from the transformed cells were seen only rarely, the detection by immunodiffusion of virion antigens in extracts of transformed cells and tumour cells was a reproducible finding (see Fig. 2). Similarly, it was possible to demonstrate the presence of viral antigens on the surface of the cell membrane by the immunoperoxidase technique, using our reference antiserum against virion antigens. Figure 2 shows unequivocal staining of the cell surface; there was probably much antigen in the cytoplasm also but immunoglobulins cannot penetrate into living cells or cells which have been fixed with glutaraldehyde.

*The response of the regional node to local subcutaneous injections of transformed cells or tumour cells*

Responses were monitored in over 20 experiments by observing the changes in the numbers and types of cells in the efferent lymph from unanaesthetized sheep for periods of up to 40 days. In general terms, the observed responses were the same as those seen after the injection of bacteria, viruses or foreign leucocytes (Hall and Morris, 1963; Hall *et al.*, 1967; Denham *et al.*, 1969; Smith and Morris, 1970) and at the peaks of the responses, *i.e.* between 100 and 200 h after stimulation, 20–40% of the lymph cells were immunoblasts. The appearance of these cells under the electron microscope was identical to that seen in previous studies (Hall *et al.*, 1967). Cytotoxic antibodies, assayed against L14 tumour cells, began to appear in the lymph at about 120 h reaching a peak value between 300 and 400 h. In spite of our attempts to localize the stimulus to the regional node, cytotoxic antibodies usually became detectable in the blood serum at about the eighth day (200 h). Antibodies to virion antigens in the lymph plasma and blood serum could not be detected by immunodiffusion, even in concentrated lymph, until 250 h after primary stimulation. Later on in the secondary response, or during the growth of some actual tumours (*q.v.*), it was usual to detect antibodies by immunodiffusion using unconcentrated lymph.

Three sheep which had undergone unilateral nephrectomy *in utero* received injections of 2–4 $\times$ $10^8$ autochthonous, transformed kidney cells and the response of the regional node was monitored as before. Although there could have been no allogeneic component to the antigenic stimulus, all 3 responses were just as vigorous as those seen in response to allogeneic transformed cells. One of these sheep received 2 further doses of autochthonous transformed cells in complete adjuvant and provided a hyperimmune antiserum (HAs), with no anti-allo component, which we used as a positive control in both cytotoxicity and immunodiffusion assays.

*The properties of anti-tumour cell antibody.*—Using rabbit serum as complement, cytotoxic antibodies were found up to titres of 1 :10,000 in the sera of sheep that had been immunized with transformed cells or tumour cells. A specimen titration curve is shown in Fig. 3. Initially we used L14 cells as targets because they had a low spontaneous release of $^{51}$Cr but when the sera were titrated at the same time against different target cells we found that the antisera had no significantly greater activity against the actual cell line used for immunization than against another. Specimen results are shown in Table I. Similar results were obtained in absorption experiments. These were done by a single absorption of the test antiserum (efferent lymph collected 18 days after the injection of 4 $\times$ $10^8$ L6 cells) on equal volumes of various cells, and then titrating the cytotoxic activity against 2 different cell lines. The results are shown in Table II. Generally, these results favour the view that the cytotoxic antibodies evoked by the injection of allogeneic tumour cells were directed principally against viral antigens on the cell surface, and although alloantibody must be assumed to be present in such sera the titres were too low to interfere significantly in the assay system used. Similarly, the Jarrett antiserum, which was produced by immunization with

TABLE I.—*Titres of Cytotoxic Antibody at Various Times in the Lymph Plasma, Efferent from the Regional Node, and the Blood Serum of Sheep which had received Subcutaneous Injections of Transformed Cells or Tumour Cells*

| | | | Efferent Lymph Plasma | | Blood Serum | |
|---|---|---|---|---|---|---|
| Sheep No. | Cells used for immunizing dose | Cells used as targets | H after immunization | Titre of cytotoxic antibody | H after immunization | Titre of cytotoxic antibody |
| GT 57 | $6 \times 10^7$ | $L_6$ | 12 | <1 : 50 | 22 | <1 : 50 |
| | fresh, | | 58 | <1 : 50 | 68 | <1 : 50 |
| | $L_6$ cells | | 152 | 1 : 10000 | 250 | 1 : 5000 |
| | | $L_{14}$ | 12 | ≤1 : 500 | 22 | <1 : 50 |
| | | | 152 | ≥1 : 2000 | 250 | ≥1 : 2000 |
| | | | 250 | ≥1 : 2000 | 960 | ≥1 : 2000 |
| GT 59 | $10^7L_6+$ | $L_6$ | 70 | <1 : 50 | 73 | <1 : 50 |
| | $10^7L_{14}+$ | | 263 | ≥1 : 5000 | 215 | 1 : 800 |
| | $10^7$SK5/FeSV | | 405 | ≥1 : 5000 | 405 | 1 : 10000 |
| GT 29 | $6 \times 10^7$ | $L_6$ | 87 | <1 : 50 | 97 | <1 : 50 |
| | SK5/FeSV | | 143 | 1 : 1000 | 143 | 1 : 500 |
| | | | 230 | 1 : 1500 | 234 | 1 : 1000 |

Cell lines $L_6$ and $L_{14}$ were established from actual tumours. Cell line SK5/FeSV was produced by transforming normal kidney cells with FeSV, *in vitro*.

≥ Indicates that specific release was still maximal at highest dilution tested; the end point would be at least 3 or 4 times greater than that indicated.

TABLE II.—*The effect of Various Absorptions on the Cytotoxic Activity in the Serum from a Sheep Immunized with L6 Cells*

| Cells used for adsorption | Cytotoxic titre against $L_{14}$ cells | Cytotoxic titre against $L_6$ cells |
|---|---|---|
| None | ≥1 : 5000 | ≥1 : 5000 |
| $L_{14}$ | <1 : 500 | < 1: 500 |
| $L_6$ | <1 : 500 | <1 : 500 |
| SK5/FeSV | <1 : 500 | <1 : 500 |
| SK5 (normal kidney) | ≥1 : 5000 | ≥1 : 5000 |

≥ Indicates that specific release was still maximal at highest dilution tested; the end point would be at least 3 or 4 times greater than that indicated.

purified virus, had a high titre against all the cell lines except normal kidney. Also, antiserum HAs, which contained no anti-allo component routinely gave titres in excess of 1: 5000 against tumour cells and transformed cells. None of the many cytotoxic sera tested had significant lytic activity against cultures of normal, untransformed kidney cells.

*Antibody class.*—Column chromatography (Williams and Chase, 1968) of cytotoxic sera yielded IgGl fractions that contained the bulk of the cytotoxic activity; IgGl is the principal complement binding IgG antibody in sheep sera, at any rate in *in vitro* systems (Feinstein and Hobart, 1969). In cytotoxic sera collected early in the response, *e.g.* lymph plasma 120–200 h after stimulation, some cytotoxic activity was present in the IgM fraction also.

*Immunodiffusion results.*—Immunodiffusion was much less sensitive than the cytotoxic assay but the results illustrate the potential complexities of the system. Although against purified, disrupted FeLV preparations the sheep antisera usually gave one major line, at least 2 other subsidiary lines were often present, and similar results were obtained when extracts of cultured tumour cells or transformed cells were used as the antigen (Fig. 1). The feline leukaemia-sarcoma viruses contain several antigenically distinct

FIG. 1.—Photographs of 2 immunodiffusion gels, (a) and (b). (a) Centre well contains hyperimmune serum (HAs) from a sheep immunized with autologous transformed cells. The top well and the right hand well contained purified, disrupted feline leukaemia virus (FeLV). The bottom well and the left hand well contained extracts from cultured $L_{14}$ and $L_6$ cells respectively. Note the general identity and the presence of 3 precipitin systems. (b) Centre well contains purified, disrupted FeLV. The top well contains peripheral lymph draining from the tumour of sheep YT 93, doubling dilutions of this material proceed in a clockwise sequence in the other 3 wells.

components and it is unlikely that the antisera would be directed against a single antigen. The sera which gave obvious precipitin lines against purified, disrupted virus, always contained substantial titres of cytotoxic activity, usually in excess of 1:1000.

*The induction of tumours in sheep.*—Injections of inducing cells were given into the lateral aspect of one of the hind legs, *i.e.* into the cannon, an area which drains principally, though not entirely, to the popliteal node. We reasoned that if there were an indwelling cannula in the efferent duct of the node, the continual loss to the sheep of specific antibody and lymphoid cells would reduce the vigour of the systemic immune response against the viral or other antigens and so encourage tumour development (cf. Hall *et al.*, 1967). All but one of the 12 sheep in this series therefore received their dose of inducing cells into the cannon and 8 of them had been provided with a chronic fistula of the efferent duct of the regional popliteal node a few days before the injection of the cells. The results of this series of experiments are summarized in Table III.

Tumours occurred in 4 of the 12 sheep. They were all poorly differentiated, unencapsulated fibrosarcomata (cf. Snyder, Theilen and Richards, 1970.) Both the primary tumours and secondary deposits were well vascularized and free from areas of cavitation and necrosis. The appearance of these tumours in electron microscope studies is shown in Fig. 4 and 5. The details of each case were as follows:

*Ewe YT 645.*—This sheep was thymectomized when it was a month old and 3 months later the thoracic duct was drained for 2 weeks so that $10^{11}$ lymphocytes were removed. Next, 180 rad of whole body irradiation was given. Two weeks later the WBC was only $1200/mm^3$ but reached $2500/mm^3$ after a further 10 days. After this the sheep remained in apparent health and gained weight normally. Two hundred days after thymectomy (*i.e.* approximately 75 days after irradiation) the total WBC was

FIG. (2a)

FIG. 2(b)

FIG. 2.—Electron micrographs × 42,000 of cultured $L_6$ cells stained with immunoperoxidase reagents. (a) Surface of $L_6$ cell exposed to a 1 in 10 dilution of goat anti-FeLV serum (Jarrett) and counter-stained with a horse anti-goat Ig serum conjugated with horseradish peroxidase. The electron dense product resulting from the interaction of peroxidase, peroxide and benzidene is localized to the cell membrane. (b) Surface of $L_6$ cell exposed to a 1 in 10 dilution of control normal goat serum before being treated with the peroxidase conjugate and benzidene reagent. No staining has occurred.

2500/mm$^3$, of which 40% were granulocytes. At this time 4 × $10^8$ L6 cells were injected into the right cannon and a tumour became palpable 14 days later. After another week, when the tumour was well established, the efferent lymphatic duct of the regional popliteal node was cannulated. The lymph was collected quantitatively for 5 days until the cannula became blocked with fibrin. The output of lymphocytes was not high, only 30 million per h, and no more than 9 % of the cells were immunoblasts. In spite of this, the blood serum and the lymph plasma showed cytotoxic activity of dilutions above 1:2000 and gave positive precipitin lines in immuno-diffusion without being concentrated. The tumour continued to grow. Twelve days later it was on the point of ulcerating through the skin so that surgical excision was an immediate necessity. Approximately 100 g (wet weight) of tumour was removed; it had penetrated into the dermis, infiltrated the tendon sheaths and was firmly attached to bone. Complete excision was thus impossible. It was expected that there would be a local recurrence of the tumour and that the skin wound would break down. This did not happen and the wound healed perfectly.

At the time of the operation approxi-

FIG. 3.—Typical titration curve obtained by measuring the release of $^{51}$Cr from prelabelled target cells exposed to rabbit complement and serial dilutions of antiserum. Two separate titrations of the same antiserum are denoted by open and closed circles. Each point represent the median result from 3 replicate assays. The end point was taken as that dilution which would cause a release of $^{51}$Cr equal to half of the observed maximum. " Specific release " refers to the excess of $^{51}$Cr released in test systems above that released in the presence of negative control sera.

mately $3 \times 10^8$ cells, prepared from the fresh tumour, were injected s.c. into the point of the right shoulder but failed to grow. These cells were viable because *in vitro* cultures were established from the same sample and grew well.

This sheep was kept to see if metastatic tumours would declare themselves; a year later the sheep was well grown and in robust health with an essentially normal blood count. No sign of tumour was detected at post mortem.

*Wether YT 93.*—The efferent duct of the left popliteal node was cannulated before $4 \times 10^8$ L6 cells were injected into the left cannon. By 120 h after the injection the basal output ($35 \times 10^6$/h) had more than trebled and 30% of the lymph cells were immunoblasts. Just before the preparation failed, at 262 h (11 days) the output was 60 million cells per h and 20% of the cells were still immunoblasts; antibody was detected by immunodiffusion in concentrates of both efferent lymph plasma and blood serum, and a tumour had just become palpable at the injection site. Five days later a punch biopsy of the tumour was taken and a peripheral lymphatic draining directly from the tumour was cannulated. This flowed for 6 days (*vide infra*). During this time the antibody levels increased rapidly; even unconcentrated peripheral lymph gave strong lines in immunodiffusion tests and by Day 40 the blood serum was specifically cytotoxic at a dilution of 1:5000. None the less, the tumour continued to grow and ulcerated through the skin 43 days after the L6 cells had been injected. The sheep was then killed and dissected. The primary tumour had a wet weight of nearly 200 g. Five satellite tumours, each about 1·5 cm in diameter, were found on the medial side of the thigh along the line of the afferent lymphatic vessel which drained to the inguinal node (Fig. 6). No deposits of tumour were found anywhere else.

Histological examination of the popliteal node draining the primary tumour and the inguinal node draining the satellite tumours showed them to have been in a hyperreactive state. Lymphatic nodules and germinal centres were abundant, the paracortices were well populated with small lymphocytes and immunoblasts and the medullary cords were replete with plasma cells. Sinus histocytosis was not conspicuous and there was no sign of either viable or aborted malignant deposits.

The general histological character of both the primary and secondary tumours was similar, as described above. Conventional

FIG. 4.—Electron micrograph × 5280 of the progressive sarcoma induced in sheep YT 645, to show the characteristic cytology. One of the tumour cells is in mitosis.

light microscopy showed no easily discernible mononuclear cell infiltrate; electron microscopy confirmed the paucity of host cells. Only occasional neutrophil granulocytes, and even fewer round cells, were seen.

The occurrence of the satellite tumours on the medial side of the thigh emphasized that a lymphatic pathway between this area and the injection site was much commoner than we had supposed. Similarly, in another sheep (GT 57), in which tumour induction had failed, we injected dye into the cannon and

FIG. 5.—Electron micrograph × 5280 of the progressive sarcoma induced in sheet YT 93. The cell with inclusion bodies in the lower part of the picture is a macrophage, but host cells of any type were relatively rare.

found a lymphatic leading to the inguinal node *via* the medial side of the thigh. Further studies revealed that this was a frequent situation and was particularly common in females where the inguinal-mammary lymph node system is larger than in males. We came to regard this lymphatic pathway as being potentially present in all sheep and it was for this reason that we included a Magellan operation with the last 3 preparations with popliteal cannulae; one of these, GT 62, developed a tumour.

FIG. 6.—Sketch diagram to show the position of primary and secondary tumours in sheep YT 93 and GT 62.

*Ewe GT 62*.—Cannulation of the popliteal efferent duct and a Magellan operation were performed on the left hind leg 3 days before $1{\cdot}4 \times 10^8$ L6 cells were injected into the cannon. Lymph flowed for 40 days and during this time a primary tumour appeared followed shortly by 2 satellite tumours in the scar of the Magellan incision. One of the satellite tumours was removed for histological study and an afferent lymphatic draining from the tumour was cannulated. Thereafter the tumours regressed completely. The salient features of the experiment are represented graphically in Fig. 7.

An interesting finding in this experiment was the slow development and low level of antibody activity. Low or delayed titres in the blood serum might have been explained, perhaps, by the quantal removal of antibody and activated cells *via* the lymphatic fistula, were it not for the fact that the lymph itself contained much less antibody than had been found in previous experiments. In spite of a vigorous immunoblast response, immunodiffusion tests of concentrated lymph did not yield positive results until 450 h. Similarly, cytotoxic activity was not detected until 300 h, and then only at dilutions of 1:100 or less. Little antibody activity was detected in blood serum until 500 h when concentrated material was just able to produce a faint line in the immunodiffusion system.

The position of the 2 satellite tumours in relation to the primary tumour is shown diagrammatically in Fig. 6; the smaller of the 2 satellite tumours was removed surgically on Day 18. Conventional histological preparations revealed an abundant infiltration of the tumour tissue with round cells which were seen to be almost entirely small lymphocytes in electron microscope studies (Fig. 8). Such a histological appearance might be expected to herald the inhibition or reversal of tumour growth and, in fact, that is what happened. By Day 22 it was obvious that the remaining tumour tissue was shrinking and by Day 40 no external trace of the tumour was visible. An examination under anaesthetic was performed but no swelling of any sort could be found and the contours of the leg were restored perfectly.

It was estimated that before tumour regression began there must have been at least 50 g of tumour tissue in the leg. While this melted away (under the onslaught of the small lymphocytes?) one might have expected some marked alteration in the flow rate or composition of the afferent or efferent lymph. However, no very significant alteration took place. In retrospect, it seems remarkable that 50 g of highly cellular and antigenic tissue could have vanished so unobtrusively.

The sheep was kept in order to see if any further disease would declare itself but after 5 months it was in perfect health and no abnormality was detected at post mortem.

*YT 170*.—This sheep, a female Downs lamb, received a deep s.c.–i.m. injection of $5 \times 10^8$ *in vitro* transformed cells (SFKI/FeSV) into the left shoulder region when it was 5 months old. No palpable tumour resulted and 7 weeks later it was explored surgically. A substantial tumour was found adhering to the deep surface of the brachiocephalic muscle and infiltrating down and around the great vessels at the root of the neck. About 30 g of tumour (judged to represent $\frac{3}{4}$ of the total) was excised; the remaining tumour was associated too closely with major blood vessels for easy removal.

A cell suspension was made from the fresh tumour and although this grew well *in vitro* an inoculum of approximately $4 \times 10^8$ cells failed to grow when injected s.c. into the left flank. Similarly, the primary tumour showed no signs of growth and at post mortem, 3 weeks later, no trace of tumour could be found anywhere in the body.

Histological examination of the tumour showed it to be the usual undifferentiated sarcoma but there was an abundant round cell infiltrate. Electron microscope studies

FIG. 7.—Graphical representation of the flow and composition of the efferent lymph from the regional popliteal node of sheep GT 62 during the induction, growth and regression of a sarcoma induced by the subcutaneous injection of allogeneic transformed cells. The dotted line joining the square points shows the flow rate of the lymph; the open histogram shows the total cell output and the blocked area shows the output of immunoblasts.

showed that these were mainly small lymphocytes but neutrophil granulocytes were plentiful also.

As with all the above tumours, a " Brij 58 " extract of the tumours was found by immunodiffusion to contain viral antigens. Samples of blood serum, collected at the time of operation and at post mortem contained no antibody that could be detected by either the cytotoxic or immunodiffusion tests. During these tests the positive controls worked perfectly and we are confident that our failure to find antibody does not represent a trivial technical mishap.

*Studies on peripheral (afferent) lymph draining directly from tumours*

Peripheral lymph draining from the tumour was collected from 3 sheep, YT 645, YT 93 and GT 62. The results are summarized in Table IV.

In general terms, the lymph from all 3 tumours was similar to that collected from the peripheral tissues of normal sheep (Hall and Morris, 1963; Morris, 1968; Smith, McIntosh and Morris, 1970*a*). The distinctive features of peripheral lymph are its low content of white cells and the presence of macrophages, which are, for practical purposes, absent from intermediate and central lymph.

The only abnormalities we detected were an increase in the percentage of immunoblasts present. This was definitely raised in YT 93 and marginally raised in YT 645 and is indicative of a cellular reaction to antigens which have entered the tissues from which the lymph is coming (Hall and Morris, 1963). An increase in immunoblasts was not found in peripheral lymph from GT 62; the increased numbers of neutrophil granulocytes in this preparation is a usual finding for the first day or two after a cannula has been inserted.

Naturally, a thorough search was made for the presence in the lymph for any malignant cells which had detached themselves from the primary tumours.

FIG. 8.—Electron micrograph × 7200 of one of the satellite deposits from the regressing sarcoma in sheep GT 62. A tumour cell can be seen inside a capillary. Several of the surrounding cells are small lymphocytes. The cytoplasmic process in the lower part of the picture probably is part of a plasma cell or plasma blast. The whole of the specimen was infiltrated with lymphoid cells.

Because the cell count of peripheral lymph is low, it is easy to deposit the cells from many ml and to resuspend them in a drop of medium so that they can be inspected directly with a × 10 objective. Very large cells, such as tumour cells, stand out prominently and, when discovered, their nature can be established by

TABLE III.—*Chronological Summary of Formal Experimental Attempts to Induce Tumours in Sheep by the Subcutaneous Injection of Cultured Allogeneic Tumour Cells, or Transformed Cells, into the lateral Aspect of the Cannon**

| Sheep No. | No. and type of cells injected | Pretreatment of sheep | Result |
|---|---|---|---|
| Ewe YT 705 | $4 \times 10^8$ SK2/ FeSV, from store | Cannulation of regional popliteal efferent duct | Lymph flowed for 45 days. No tumour developed. |
| Ewe YT 645 | $4 \times 10^8$ L6, from store | Thymectomy, whole body irradiation. Thoracic duct drainage | Tumour palpable 15 days after injection of cells. Progressive tumour growth necessitated surgical excision on Day 33, sheep alive and well 1 year later. |
| Wether YT 93 | $4 \times 10^8$ L6, from store | Cannulation of regional popliteal efferent duct | Tumour palpable at Day 13. Progressive growth with ulceration and the appearance of five satellite tumours in the thigh. Sheep killed on Day 43. |
| Wether YT 163 | $4 \times 10^8$ L6, from store | None | No tumour. |
| Ewe YT 141 | $1 \times 10^8$ L6, half of them fresh | Cannulation of regional popliteal efferent duct | Lymph flowed for 38 days. No tumour developed. Post mortem (PM) on Day 38 normal. |
| Wether YT 491 | $3{\cdot}5 \times 10^8$ L6, from store | Cannulation of regional popliteal efferent duct | Lymph flowed for 64 days. No tumour developed. |
| Wether GT 57 | $6 \times 10^7$ fresh L6 | Cannulation of regional popliteal efferent duct | Lymph flowed for 15 days. No tumour developed. PM on Day 30, normal. |
| Week old lamb | $7 \times 10^7$ fresh L6 | None | No tumour. The lamb grew normally. PM on Day 200 normal. |
| Ewe GT 29 | $6 \times 10^7$ fresh SKS/FeSV | Cannulation of regional popliteal efferent duct. Magellan operation. | Lymph flowed for 15 days. No tumour developed. PM at 150 days normal. |
| Ewe GT 59 | $10^8$ L6, $10^8$ L14, $+10^8$ SKS/FeSV all from store | Cannulation of regional popliteal efferent duct. Magellan operation. | Lymph flowed for 23 days. No tumour developed. PM at 150 days normal. |
| Ewe GT 62 | $7 \times 10^7$ fresh L6, $+7 \times 10^7$ stored L6 | Cannulation of regional popliteal efferent duct. Magellan operation. | Lymph flowed for 40 days. Tumour palpable on Day 13. Secondary deposits seen on skin wound on Day 15. Tumours started to regress on Day 22 and had gone by Day 40. Sheep alive and well 5 months later. PM normal. |
| Ewe YT 170 | $5 \times 10^8$ SFKI/ FeSV *injected s.c. into the left shoulder | None | Tumour revealed by surgical exploration 7 weeks after injection. Subtotal excision. Complete regression of remaining tumour confirmed at post mortem. |

* L6 and L14 were cell lines derived from actual tumours. Cells designated " FeSV " were foetal cells transformed *in vitro*.

direct examination with an $\times$ 100 phase objective. In this way it is possible to monitor the lymph for the presence of tumour cells without too much difficulty. Nonetheless, only 2 tumour cells were seen; both were present in peripheral lymph cells from YT 93. It was concluded that in this particular tumour system the spontaneous shedding of intact tumour cells into the lymph stream occurs only infrequently. Incidentally, tumour cells were never seen in the regional efferent lymph from either tumour bearing sheep or from sheep which had received injections of tumour cells.

Afferent popliteal lymph from normal sheep of the type used in these experiments normally flows at between 1 and 2 ml/h. The fact that lymph flow from the afferent preparation in YT 645 averaged 6 ml/h and sometimes flowed at as much as 9 ml/h indicated to us that much of the lymph was being generated in the additional capillary bed provided by the tumour tissue. For this reason we took the opportunity to inject $^{125}$I-BSA intravenously into this sheep so that we could investigate the function of the tumour capillary bed in terms of the partitioning of the labelled protein between the intra-

TABLE IV.—*The flow and Composition of Peripheral Lymph, Afferent to the Popliteal Node, Draining from Established Tumours of the Lower Part of the Leg (Cannon) in Sheep*

| Sheep no. | Time after inj. of inducing cells at which cannln. performed | Flow rate and duration of preparation | Total white cells per $mm^3$ | Differential white cell count (%) | | | | Protein conc. as % of blood plasma value | Antibody titre | |
|---|---|---|---|---|---|---|---|---|---|---|
| | | | | Neut. Polys. | Small lymph-ocytes | Immuno blasts | Macro phages | | I.D. | Cytotoxic assay |
| YT 645 (Tx) | 21 days | 6·0 ml/h 14 days | 1200 | 5 | 60 | 6 | 29 | 27 | 1 : 4—1 : 8 | 1 : 2000 |
| YT 93 | 17 days | 1·5 ml/h 6 days | 500 | 6 | 66 | 18 | 10 | 22 | 1 : 8—1 : 16 | 1 : 4000 |
| GT 62 | 20 days | 1·0 ml/h 2 days | 700 | 15 | 78 | 2 | 5 | 24 | just +ve after 20 fold conc. of globulin | ± equi-vocal |

FIG. 9.—The specific radioactivities of plasma proteins in blood ●——●; afferent popliteal lymph ▲——▲, and efferent prefemoral lymph △——△, after the intravenous injection of $^{125}$I-BSA at time zero. The results of 2 experiments are shown. The graph on the left shows the results obtained from a normal sheep where equilibration between the blood and afferent lymph occurred at 9 h. The graph on the right shows the results obtained from a tumour bearing sheep where the afferent lymph, coming directly from the tumour, equilibrated with the blood after 7 h.

vascular (blood plasma) and the extravascular (lymph plasma) plasma protein pools. The experiment was carried out as described above; the results, including those from a control sheep, are shown in Fig. 9. It can be seen that equilibration of labelled albumin between the blood and the afferent lymph (*i.e.* the tissue fluid of the tumour) had taken place within 7 h. The corresponding result from the experiment on the normal sheep was 9 h. In both sheep the prefemoral lymph took much longer to equilibrate but this probably reflects the fact that the prefemoral node drains a larger area of skin with a thick, adipose subcuticulum and so contains a much larger pool of plasma protein than the extremity of the leg. These results should not be overinterpreted but they suggest that the permeability of the capillaries in the tumour is at least as great as that of those in normal tissue. This finding is consistent with the abundance of antibody that was present in the afferent lymph of YT 645 and YT 93. Clearly the antibody has no difficulty in escaping from the blood into the interstitial fluid of the tumour.

### DISCUSSION

The ability of the FeSV system to induce tumours in heterospecies as diverse as dogs, rabbits and monkeys (Snyder and Theilen, 1969; Theilen *et al.*, 1969; Deinhardt *et al.*, 1970) and, more recently, in sheep (Theilen, 1971; Theilen *et al.*, 1974) and rats (Maruyama, Wagner and Domchowski, 1973) makes possible the study of experimentally induced tumours in large mammals. The object of the present work was to study the responses of the regional lymphatic system of sheep to the onslaught of solid, malignant tumours, rather than to study viral oncogenesis *per se*. Nonetheless, our findings cannot be interpreted easily without reference to the antigenic structure of tumours that have been induced with feline oncorna viruses (FOV). Reviews of this large and sometimes controversial topic are available (Essex, 1974; Jarrett, 1975) but many uncertainties exist. In earlier work on tumours induced with oncorna viruses it seemed that the serological responses were directed principally against virus material that had become incorporated in the cell membrane (Hardy *et al.*, 1969; Sibal *et al.*, 1970; Riggs, 1971; Riggs *et al.*, 1973). More recently an additional antigen, designated feline-oncorna virus associated antigen (" FOCMA ") has been reported (Essex *et al.*, 1971, *a,b*). This antigen is believed not to be part of the virion and is said to evoke antibodies which are associated with tumour regression or a favourable prognosis in cats infected with the feline leukaemia-sarcoma virus (Essex, 1974; Jarrett, 1975). Although we do not wish necessarily to exclude the presence of such a neoantigen, we could find no serological evidence for its existence in the sheep system. We conclude, at any rate for the purposes of this discussion, that the humoral responses we detected can be accounted for in terms of immunoglobulins with specificity for proteins and glycoproteins of the virus. Immunodiffusion showed up to 3 distinct antigen–antibody systems. Probably the major one would involve the gs-interspecies antigen (Schäfer *et al.*, 1971) sometimes referred to as gs-3 (Geering, Aoki and Old, 1970) which is now known to be present on a polypeptide with a molecular weight approaching 30,000 which is the major structural protein of the virion (Strand and August, 1974; Jarrett, 1975).

In general, the responses of the regional nodes to the antigenic stimuli provided by tumour cells were vigorous but normal and provide no *prima facie* suggestion that the virus or its products exerts the immunosuppressive effects that have been encountered in cats with systemic FeLV infections (Jarrett, 1975).

We were surprised by the fact that the responses to allogeneic transformed cells or tumour cells were no greater than those to syngeneic transformed cells and that no unequivocal evidence of antibodies directed against allogeneic histocompatibility antigens could be found. We know that sheep will make easily detectable lysins after a primary challenge with allogeneic lymphocytes (Scollay, Lafferty and Poskitt, 1974). Presumably, in the present experiments some alloantibodies were formed but were present only in much lower titre than the antibodies directed against the viral proteins on the cell membrane. Conceivably, the presence of these relatively strong viral antigens " competed " successfully in the induction phase against the allogeneic histocompatibility antigens which, in sheep, tend to be relatively feeble immunogens unless they are presented on viable lymphoid cells (Scollay *et al.*, 1974).

The question of the actual tumours in sheep now arises. Of the 4 tumours described, 3 were induced with L6 cells which display a male karyotype. Two of these tumours arose in female hosts but only one, growing in YT 645, gave rise to a successful *in vitro* culture. Unfortunately, this line was destroyed by a pseudomonas infection before a karyotypic analysis could be performed, so in none of the cases have we direct proof that the tumours were of host origin. However, our previous studies on lambs (Theilen *et al.*, 1974) showed that wherever an interpretable karyotypic analysis was possible the tumours were always found to be of host origin. This fact, together with the well documented potential of the FeSV system for inducing syngeneic tumours in heterospecies, make it virtually certain that the tumours arose from host cells that were transformed by virus present in the cells of the inducing inoculum.

We do not know why only a minority of our test sheep developed tumours. It seemed that the breed and sex of the sheep were not important factors in tumour induction. Tumours occurred in a Clun Forest ewe (YT 645), a small crossbred mountain wether (YT 93) and in 2 Southdown ewes (GT 62 and YT 170). Probably the most important single factor in tumour induction was the administration of a sufficiently large dose of transformed cells or tumour cells. However, in order to attain high cell numbers

(up to $5 \times 10^8$) the use of stored cells was inevitable but we have no evidence that stored cells are less able to induce tumours than fresh cells.

That the first case of successful tumour induction in a grown sheep occurred in YT 645, the ewe that had been deliberately immunosuppressed, seems logical but may be fortuitous. Even total thymic deprivation dose not impair homograft rejection in sheep (Cole and Morris, 1971), although it may favour the growth of an established tumour (Theilen *et al.*, 1974) and, in spite of the immunosuppressive procedures, this sheep produced abundant antibody. Certainly, in the second case of successful tumour induction (YT 93) there was no immunosuppression other than that provided by the cannulation of the regional node, which in any case did not seem to delay the appearance of humoral antibody in the blood vascular compartment. Nonetheless, the same dose of the same cells given in the same way to its uncannulated twin (YT 163) failed to produce a tumour. Similarly, GT 62 received no immunosuppression, other than cannulation, and although it developed a tumour it was able to reject it. This result does not accord well with the idea of immunosuppression as an essential prerequisite for tumour induction, especially as YT 170, the last case of successful induction, received no experimental immunosuppression at all. Thus, although cannulation of the regional node provides a useful way of monitoring the immune response, there are insufficient data to permit a conclusion about its role, if any, in immunosuppression and tumour induction. What did emerge was the fact that the lymph from the site on the lateral aspect of the leg that received the cell inocula, sometimes drained as much to the mammary-inguinal nodes as to the popliteal. In ewes that had had an indwelling cannula in the efferent duct of the popliteal node for more than 10 days, we found nearly always a well developed collateral, afferent lymphatic running from the lower leg to the mammary node, in spite of previous lymphangiectomy. In theoretical considerations of immune responses in cannulated sheep it has been assumed that material injected subcutaneously into the leg must go exclusively to the popliteal node (McConnell, Lachman and Hobart, 1974); obviously, this assumption is quite wrong in any general sense.

In spite of many uncertainties about the immunology of these tumours, it does seem that the responses to tumours which regressed were qualitatively different from those to tumours which grew progressively. Baldly stated, regression occurred when tumours were infiltrated with lymphocytes and began at times when cytotoxic and antiviral antibody activity was low or absent, whereas progression occurred in the absence of a significant host cell infiltrate and in the face of high titres of antibodies in the blood and in the interstitial fluid of the tumours themselves. *Prima facie*, this seems to be a simple restatement of the classic skin homograft model and also calls to mind the suggestion of Parish (1971) that there is a reciprocal relationship between humoral and cell mediated immunity. Clearly, the situation is complicated and one could speculate at length about the blocking effects of antibody, antigen or antigen–antibody complexes etc., but in the absence of further data such discussion is out of place and is available elsewhere (Currie, 1974). However, some points of detail remain. For example, although the progressive tumour in YT 645 was not excised totally the tumour did not recur and in spite of, or perhaps because of, a high antibody titre a substantial tumour autograft was rejected and distant metastases did not occur. Clearly, host resistance of some sort was by no means absent. Also, ewe GT 62 in which the tumours regressed, did manage in the end to generate a small amount of circulating antiobdy. However, it is important to stress that the failure of this sheep to produce high antibody titres cannot be accounted for simply in terms of B cell anergy. The immunoblast response in the efferent lymph was vigorous and the xenogeneic transfer test (Hall *et al.*, 1971) showed that these cells were generating abundant immunoglobulin, albeit of a nonspecific or low affinity nature. Proponents of the FOCMA concept (*vide supra*) might suggest that tumour regression was brought about by an antibody to a neoantigen that would not show up in the ID system and would not necessarily bind rabbit complement. We have attempted some preliminary experiments to detect such antibodies directly, by *e.g.* mixed haemagglutination, but so far have had no success. Also, if substantial amounts of a noncomplement binding or " blocking " antibody were to be evoked by the progressive tumours, obvious prozone effects in the titration of highly cytotoxic antisera might have been observed; they rarely were, cytotoxic antisera from tumour bearing sheep were still optimally lytic at very high concentrations.

There remains the question of metastasis. Apparent metastatic deposits occurred in 2 sheep, YT 93 and GT 62. The secondary deposits in YT 93 were relatively small in relation to the primary and probably were genuine metastases that arose relatively late in the disease process. However, the 2 satellite tumours that occurred in GT 62 declared themselves soon after the primary tumour was evident and may well have originated from some of the primary inoculum which leaked out of disrupted lymphatics in the wound caused by the Magellan operation. We found no example of distant, haematogeneous metastasis in any of the sheep, even though the electron microscope studies showed that the malignant cells can get into blood vessels.

Other properties of the blood vessels in the tumours were revealed by experiments in which peripheral lymph coming from the tumours was collected. When there was a substantial titre of antibody in the blood plasma, the antibody found no difficulty in transuding through the capillaries in the tumour and entering the interstitial fluid. The extravasation of antibodies into the peripheral tissue fluid of sheep have been investigated previously (Hall *et al.*, 1969) and there is no doubt that 7S antibodies can penetrate quite easily through the wall of normal capillaries. In this respect the capillaries in the tumours were just as permeable as those in normal tissue; indeed the equilibration studies with $^{125}$I-albumin suggested that they might be slightly more permeable than normal but there was no evidence that the capillaries of the tumours were grossly "leaky". Neither the protein nor the red cell content of peripheral lymph coming from the tumours was increased; this could not have been the case if the capillary bed in the tumours had been grossly hyperpermeable. Similarly, the number of white cells in the peripheral lymph were within usual limits (Smith *et al.*, 1970*a*) indicating that the transmigration of monocular leucocytes from blood to lymph was proceeding normally. It might have been expected that a demonstrably antigenic tumour would behave like other antigen depots and provoke characteristic changes in the cellular composition of the afferent lymph (Hall and Morris, 1963; Smith *et al.*, 1970*b*). Apart from a slight increase in the number of immunoblasts, we saw no sign in the peripheral lymph of a vigorous immunological or inflammatory reaction going on in the tumours. On the other hand, the idea (Alexander and Hall, 1970) that solid, sarcomatous tumours may escape immune destruction because defective functioning of the tumour capillaries prevents the influx of cellular and humoral factors is obviously not applicable to these sheep tumours.

Another point of interest was the infrequency with which we found tumour cells in the afferent lymph. The only definite finding of lymph-borne tumour cells was in YT 93 where secondary deposits occurred along the collateral afferent lymphatic leading to the inguinal node. Just why successful secondary deposits occurred in this site while the actual node which this lymphatic supplied remained free from tumour must remain a matter for speculation.

Although this study may have produced more questions than answers, it nonetheless has provided us with an immunogenic and potentially metastatic tumour system in sheep. This will make possible a direct investigation of the relative cytotoxic activities of cells and humoral factors in the various tissue fluids of a large mammal throughout the course of the malignant process.

This study was supported by a specific project grant (B973/468) from the Cancer Research Campaign. While working in England Professor Theilen was supported by a Fellowship from the New York Cancer Research Foundation and U.S. Public Health Service Grant No. IRO ICA 14546–01.

We acknowledge gratefully the technical assistance of Dr D. J. Glover, Mrs Ann Pendry, Miss Pat Beardall, Miss Alina Puczylowska and Mrs Kate Steele. We thank Professor M. J. Peckham and the staff of the Radiotherapy Department, Royal Marsden Hospital, Sutton, for carrying out the whole body irradiation procedures, and Professor W. H. F. Jarrett of the Department of Veterinary Pathology at the University of Glasgow for his advice as well as for his gift of antiserum.

### REFERENCES

Alexander, P. & Hall, J. G. (1970) The Role of Immunoblasts in Host Resistance and Immunotherapy of Primary Sarcomata. *Adv. Cancer Res.*, **13**, 1.

Cole, G. J. & Morris, B. (1971) Homograft Rejection and Hypersensitivity Reactions in Lambs Thymectomized *in utero*. *Aust. J. exp. Biol. med. Sci.*, **49**, 75.

Currie, G. A. (1974) *Cancer and the Immune Response*. London: Edward Arnold. p. 60.

Deinhardt, F., Wolfe, L. G., Theilen, G. H. & Snyder, S. P. (1970) ST-feline Fibrosarcoma Virus: Induction of Tumors in Marmoset Monkeys. *Science N.Y.*, **167**, 881.

Denham, S., Hall, J. G., Wolf, A. & Alexander, P. (1969) The Nature of the Cytotoxic Cells in Lymph following Primary Antigenic Challenge. *Transplantation*, **7**, 194.

Essex, M. (1974) Th Immune Response to Oncornavirus Infections. In *Viruses, Evolution and Cancer*. Eds. E. Kerstak, and K. Maramarosch. New York and London: Academic Press.

Essex, M., Klein, G., Snyder, S. P. & Harrold, J. B. (1971*a*) Antibody to Feline Oncornavirus-associated Cell Membrane Antigen in Neonatal Cats. *Int. J. Cancer*, **8**, 384.

Essex, M., Klein, G., Snyder, S. P. & Harrold, J. B. (1971*b*) Correlation between Humoral Antibody and Regression of Tumours Induced by Feline Sarcoma Virus. *Nature, Lond.*, **233**, 195.

Feinstein, A. & Hobart, M. J. (1969) Structural Relationship and Complement Fixing Activity of Sheep and other Ruminant Immunoglobulin G Subclasses. *Nature Lond.*, **223**, 950.

Geering, G., Aoki, T. & Old, L. J. (1970) Shared Viral Antigen of Mammalian Leukaemia Viruses. *Nature Lond.*, **226**, 265.

Hall, J. G. (1967) A Method for Collecting Lymph from the Prefemoral Lymph Node of Unanaesthetised Sheep. *Quart. J. exp. Physiol.*, **52**, 200.

Hall, J. G. (1971) The Lymph-borne Cells of the Immune Response: a Review. *The Scientific Basis of Medicine Annual Reviews*. London: Athlone Press.

Hall, J. G. & Morris, B. (1963) The Lymph-borne Cells of the Immune Response. *Quart. J. exp. Physiol.*, **48**, 235.

Hall, J. G., Morris, B., Moreno, G. D. & Bessis, M. C. (1967) The Ultrastructure and Function of the Cells in Lymph following Antigenic Stimulation. *J. exp. Med.*, **125**, 91.

Hall, J. G., Parry, D. M. & Smith, M. E. (1971) Antibody Production by Lymph-borne Immunoblasts following Subcutaneous Injection into Xenogeneic Recipients. *Immunology*, **20**, 625.

Hall, J. G., Smith, M. E., Edwards, P. A. & Shooter, K. V. (1969) The Low Concentration of Macroglobulin Antibodies in Peripheral Lymph. *Immulology*, **16**, 773.

Hardy, W. D. (1971) Immuno-diffusion Studies of Feline Leukemia and Sarcoma. *J. Am. vet. med. Ass.*, **158**, 1060.

Hardy, W. D., Geering, G., Old, J. L., De Harven, E., Brodey, R. S. & McDonough, S. (1969) Feline Leukemia Virus: Occurrence of Viral Antigen in the Tissues of Cats with Lymphosarcoma and Other Diseases. *Science N.Y.*, **166**, 1019.

Jarrett, W. H. F. (1975) Cat Leukemia and its Viruses. *Adv. Cancer res.* In the press.

Lascelles, A. K. & Morris, B. (1961) Surgical Techniques for the Collection of Lymph from Unanaesthetised Sheep. *Quart. J. exp. Physiol.*, **46**, 199.

McConnell, I., Lachman, P. J. & Hobart, M. J. (1974) Restoration of Specific Immunological Virginity. *Nature, Lond.*, **250**, 113.

Macpherson, I. & Bryden, A. (1972) Small Scale Replicate Animal Cell Cultures Suitable for Studies involving Radioisotopes. *Lab. pract.*, **21**, 112.

Maruyama, K., Wagner, S. H. & Domschowki, L. (1973) Sarcomas Induced in Rats by Feline RNA Virus. In *Comparative Leukaemia Research*. Eds. Y. Ito, and R. M. Dutcher. Basel: Karger and Tokyo: University of Tokyo press. p. 93.

MORRIS, B. (1968) Migration intratissulaire des lymphocytes du mouton. *Nouv. Rev. franc. d'Hémat.*, **8**, 525.

NAKANE, P. K. & KAWAOI, A. (1974) Peroxidase Labelled Antibody: a New Method of Conjugation. *J. Histochem. Cytochem.*, **22**, 1084.

PARISH, C. R. (1971) Immune Responses to Chemically Modified Flagellin. II. Evidence for a Fundamental Relationship between Humoral and Cell Mediated Immunity. *J. exp. Med.*, **134**, 21.

RIGGS, J. L. (1971) An Immuno-fluorescence Test for Feline Leukemia and Sarcoma Virus Antigens and Antibodies. *J. Am. vet. Med. Ass.*, **158**, 1085.

RIGGS, J. L., OSHIRO, L. S., DEE, O. N. T. & LENETTE, E. H. (1973) Prevalence of Type-C Virus and Antibodies in Normal Cats and Cats with Neoplasia. *J. natn. Cancer Inst.*, **51**, 449.

SCHÄFER, W., LANGE, J., BOLOGNESI, D. P., DENORONHA, F., POST, J. E. & RICKARD, C. G. (1971) Isolation and Characterisation of Two Group-specific Antigens from Feline Leukaemia Virus. *Virology*, **44**, 73.

SCOLLAY, R. G., LAFFERTY, K. J. & POSKITT, D. C. (1974) Allogeneic Stimulation Modulates the Strength of Transplantation Antigen. *Transplantation*, **18**, 6.

SHNAIN, A. H., KASSAI, T. JABBIR, M. H., KADHIM, J. K. & ALTAIF, K. I. (1973) Cannulation of the Thoracic Duct in Sheep. *Vet. Rec.*, **92**, 499.

SIBAL, L. R., FINK, M. A., PLATA, E. J., PHOLER, B. E., NORONHA, F. & LEE, K. Y. (1970) Methods for the Detection of Viral Antigen and Antibody to a Feline Leukemia Virus. *J. natn. Cancer Inst.*, **45**, 607.

SMITH, J. B. & MORRIS, B. (1970) The Response of the Popliteal Lymph Node of the Sheep to Influenza Virus. *Aust. J. exp. Biol. med. Sci.*, **48**, 33.

SMITH, J. B., MCINTOSH, G. H. & MORRIS, B. (1970*a*) The Traffic of Cells through Tissues: a Study of Peripheral Lymph in Sheep. *J. Anat.*, **107**, 87.

SMITH, J. B., MCINTOSH, G. H. & MORRIS, B. (1970*b*) The Migration of Cells through Chronically Inflammed Tissues. *J. Path.*, **100**, 21.

SNYDER, S. P. & THEILEN, G. H. (1969) Transmissible Feline Fibrosarcoma. *Nature, Lond.*, **221**, 1074.

SNYDER, S. P., THEILEN, G. H. & RICHARDS, W. P. C. (1970) Morphological Studies on a Transmissible Feline Fibrosarcoma. *Cancer Res.*, **30**, 1658.

STRAND, M. & AUGUST, J. T. (1974) Structural Proteins of Mammalian Oncogenic RNA Viruses: Multiple Antigenic Determinants of the Major Internal Proteins and Envelope Glycoproteins. *J. Virol.*, **13**, 171.

THEILEN, G. H. (1971) Continuing Studies with Transmissible Fibrosarcoma Virus in Fetal and Newborn Sheep. *J. Am. vet. med. Ass.*, **158**, 1040.

THEILEN, G. H., HALL, J. G., PENDRY, A., GLOVER, D. J. & REEVES, B. R. (1974) Tumours Induced in Sheep by Injecting Cells Transformed *in vitro* with Feline Sarcoma Virus. *Transplantation*, **17**, 152.

THEILEN, G. H., HOKAMA, Y., MANNING, J. S. & CALLAWAY, E. (1973) Heterospecies Infectivity of FeSV: Neoplasms in Sheep Fetuses and Lambs by Inoculation of FeSV-transformed Sheep Cells. In *Possible Episomes in Eukaryotes*. Fourth Lepetit colloquim. North Holland: American Elsevier. p. 109.

THEILEN, G. H., SNYDER, S. P., WOLFE, L. G. & LANDON, J. C. (1969) Biological Studies with Viral Induced Fibrosarcomas in Cats, Dogs, Rabbits and Non-human Primates. In *Comparative Leukaemia Research*. Ed. R. M. Dutcher. Basel: Karger. p. 393.

WEBSTER, R. G., LAVER, W. G. & FAZEKAS DE ST GROTH, A. E. (1962) Methods in the Immunochemistry of Viruses. 3. Simple Techniques for Labelling Antibodies with $^{131}$I and $^{32}$S. *Aust. J. exp. Biol. med. Sci.*, **40**, 321.

WILLIAMS, C. A. & CHASE, M. W. (1968) *Methods in Immunochemistry*, vol. 2. New York and London: Academic Press. p.135